# Trauma Exposure Response: How Secondary Trauma Affects Personal and Professional Life

**DOI:** 10.15766/mep_2374-8265.11192

**Published:** 2021-11-22

**Authors:** Kristin M. Jacob, Nichole Lambert

**Affiliations:** 1 Medical Director, Office of Physician and APP Fulfillment, Internal Medicine-Pediatrics, Spectrum Health/Michigan State University; 2 Behaviorist/Assistant Program Director of Family Medicine Residency, Spectrum Health/Michigan State University

**Keywords:** Trauma Exposure Response, Secondary Trauma, Compassion Fatigue, Physician, Well-Being/Mental Health, Program Evaluation

## Abstract

**Introduction:**

Well-being, both psychological and emotional, is crucial to the development of the competent, caring physician. The ACGME calls on sponsoring institutions to educate learners about topics related to well-being. Trauma exposure response, also known as secondary trauma, is a common phenomenon experienced by physicians. It is important to recognize and mitigate the effects of trauma exposure response, as it can have profound effects on personal and professional lives. We found no *MedEdPORTAL* resources on trauma exposure response or secondary trauma that include physicians as the audience.

**Methods:**

This 1-hour, interactive session was developed to embed a wellness program into protected time for residents and fellows across the institution. The session was led by a faculty member and consisted of an interactive presentation and a small-group discussion.

**Results:**

Twenty-eight of 32 programs at our institution participated in the sessions. This included a total of 292 residents and fellows to whom this session was offered. The session was successful in meeting the educational objectives and was rated as valuable or extremely valuable by most residents and fellows. Trainees appreciated protected time for this discussion and valued the opportunity to have open, honest conversations with their colleagues.

**Discussion:**

This effective session delivered meaningful content about trauma exposure response and reviewed coping strategies. Institutional support of protected time was a success factor. The sessions were well received by residents and fellows and can be used across disciplines.

## Educational Objectives

By the end of this activity, learners will be able to:

1. Define the concepts of secondary trauma and trauma exposure response.
2. Describe the signs of trauma exposure response.
3. Demonstrate techniques that can be applied to cope with trauma exposure response.

## Introduction

Physician well-being is an important initiative of the ACGME since well-being, both psychological and emotional, is crucial to the development of the competent, caring physician. This has been supported by the development of the Clinical Learning Environment Review (CLER) pathways^1^ and the common program requirements on well-being.^2^ CLER has six focus areas: patient safety, health care quality, teaming, supervision, well-being, and professionalism. The focus areas promote improvements to build an optimal clinical learning environment. ACGME program requirement VI.C.1. states that the responsibility of the program, in partnership with the sponsoring institution, to address well-being must include “policies and programs that encourage optimal resident and faculty member well-being” and “attention to resident and faculty member burnout, depression, and substance abuse.”^3^

At our institution, this session is part of a 3-year longitudinal curriculum that is built into didactics for all programs. The additional topics included in this curriculum are (1) physician depression and suicide, (2) physician impairment, (3) burnout, and (4) resilience strategies. These topics align with the ACGME common program requirements and include relevant content and discussion topics recommended by our Resident and Fellow Wellness Committee. An embedded longitudinal wellness curriculum is one of multiple programs in place to support the well-being of residents and faculty at our institution.

The key drivers of burnout include workload and job demands, control and flexibility, efficiency and resources, organizational culture and values, social support and community at work, work/life integration, and meaning in work.^4^ Many of these drivers will only be improved with high-level system change, and organizations that are committed to improving physician well-being must invest in system-level interventions. Although there is value to personal resilience and individual-level interventions, reducing burnout requires organizational-directed approaches.^5^ Embedding wellness efforts into the infrastructure of training programs is one system-level intervention to start approaching physician burnout, building community, and fostering open and honest conversations to address the unique stresses of trainees.

Forty-four percent of all physicians report symptoms of burnout.^6^ Burnout can lead to personal repercussions including depression, suicide, and substance abuse. In 2017, 42% of physicians screened positive for symptoms of depression.^6^ Burnout can also lead to professional repercussions of increased turnover, decreased productivity, decreased patient satisfaction, and decreased quality of care. Burnout is a pathologic condition that develops in response to prolonged occupational stressors and is characterized by three dimensions: (1) emotional exhaustion, (2) depersonalization, and (3) lack of personal achievement.

A unique aspect of the medical profession is exposure to trauma. This may include either primary or secondary exposure to trauma. Secondary trauma is the emotional distress that results when an individual hears about the firsthand trauma experiences of another. Secondary traumatic stress is considered a form of post-traumatic stress disorder caused by experiencing repeated or extreme exposure to adverse details of a traumatic event. Burnout and secondary traumatic stress have both been recognized as consequences of extreme demands on human service professionals. Secondary traumatic stress differs from burnout in that it can result from a single exposure to trauma versus the repeated occupational stress exposure that is characteristic of burnout. One meta-analysis indicated strong associations between job burnout and secondary traumatic stress.^7^ It remains to be clarified if there is a unidirectional or bidirectional causal relationship between burnout and secondary traumatic stress,^8^ but secondary traumatic stress can contribute to the emotional exhaustion and depersonalization components of burnout.

Over time, multiple terms have been used to approach the concept of secondary trauma. This session focuses on defining trauma exposure response, which is the transformation that takes place as a result of exposure to the suffering of others. This is a concept that many physicians, particularly trainees, can relate to and observe in themselves as they progress through medical education. This session provides a facilitated discussion to recognize and mitigate trauma exposure response. It is important to recognize and mitigate the effects of trauma exposure response as it can have profound effects on personal and professional lives.

A search for the terms *trauma* and *secondary trauma* in *MedEdPORTAL* displayed many publications related to clinical trauma and some publications on trauma-informed care toward the patient. One workshop, “Difficult Conversations After Resuscitation in Trauma: Video Education E-Module,”^9^ discussed the challenges of conversations with a patient's family members. It also included objectives on the value of self-reflection and debriefing after a traumatic event. A search for the term *compassion fatigue* resulted in one applicable curriculum: “Building Team Resilience and Debriefing After Difficult Clinical Events: A Resilience Curriculum for Team Leaders.”^10^ The objectives highlighted individual experiences with difficult clinical events as well as identifying healthy and unhealthy coping behaviors. There were no publications that discussed secondary trauma or trauma exposure response.

This session on trauma exposure response is novel in that it presents important content in an engaging and applicable way. This experience could be applied to both trainees and faculty across the health professions. This session is intended to be built into residency didactics and facilitated by a faculty member who is engaged and willing to share personal stories of vulnerability. To be vulnerable is to be emotionally available to our colleagues, requiring compassion for ourselves and others. Being vulnerable is necessary for building meaningful relationships. In providing space and time for trainees and attendings to open up and connect, this session can also help build community among participants.

## Methods

This session was designed as a 1-hour, in-person or virtual, facilitated discussion. The session included introductory didactic material that defined trauma exposure response, time for self-reflection about one's own signs of trauma exposure response, and small-group discussion focused on strategies to mitigate the effects of trauma exposure response.

The session was part of our institution's wellness curriculum. Since 2015, our institution has required that each residency and fellowship program protect 1 hour, two times per year, to talk about wellness-related curricula. The curriculum was developed and vetted by the Resident and Fellow Wellness Program and aligns with ACGME common program requirements. Each program had a designated faculty wellness champion who facilitated the session for the trainees. Representatives from our employee assistance program were also available to cofacilitate or attend the session to offer support during the sensitive discussion.

The topic of trauma exposure response was identified as an impactful subject by our Resident and Fellow Wellness Committee and based on feedback from participants in our resident debrief programs. Additionally, the session was developed during the time of the COVID-19 pandemic, when the concept of secondary trauma was especially relevant. Content was developed from a review of a large body of evidence around secondary trauma and burnout. The book *Trauma Stewardship* by Laura van Dernoot Lipsky was also a source of reference for the development of this session.^11^

In the fall of 2020, each of the 32 ACGME-accredited programs at our institution was asked to protect 1 hour of didactic time for this wellness session. Some smaller fellowship programs joined other programs for this session. Historically, these sessions have been held in person, in groups ranging from four to 40 learners, based on the size of the program. For 2020, the content was designed to be held socially distanced in person or virtually.

Each faculty wellness champion was supported by the program coordinator in scheduling the didactic session as well as printing materials, if applicable. A detailed facilitator guide was sent to the faculty wellness champion before the session. This was accompanied by the PowerPoint presentation, discussion questions, and evaluation materials. In the past, an orientation had been held for the faculty wellness champions to prepare them to lead the session. This was not held in 2020, as most faculty wellness champions were familiar with the process of the wellness session and discussion.

### Target Audience

The target audience was residents and fellows.

### Logistics

This session was built into the didactic time for residents and fellows. Each program was asked to protect 1 hour during the fall of 2020. Program coordinators helped direct the trainees' and faculty wellness champions' schedules to find an ideal time. Representatives from the employee assistance program were invited to attend if preferred by the program. Faculty wellness champions were presented with the facilitator guide (Appendix A) and other materials before the session and were encouraged to review them. Sessions were held as socially distanced, in-person didactics or via a virtual platform. Zoom was recommended as a virtual platform due to its breakout group capability. The number of participants in the various groups ranged from four to 40 trainees.

### Time Breakdown

• 5 minutes: introductions and gratitude.
• 15 minutes: interactive presentation.
• 25 minutes: small-group discussion.
• 10 minutes: report out.
• 5 minutes: closing and resource review.

The sessions began with the faculty wellness champion introducing themself. In smaller groups, it was encouraged that all participants introduce themselves and share something they were grateful for to set the tone for the session. Subsequently, the faculty wellness champion was encouraged to introduce the topic for the session and how trauma exposure is particularly relevant during the COVID-19 pandemic. In-person teams were encouraged to form socially distanced small groups.

The PowerPoint presentation (Appendix B) was then reviewed. The learning objectives were also reviewed. This was followed by defining and comparing the multiple terms related to the concept of trauma exposure. It was pointed out that this discussion would mainly focus on trauma exposure response, which is the transformation that takes place within us because of exposure to the suffering of others. Statistics related to secondary traumatic stress and burnout were reviewed. This was followed by a review of the risk factors for secondary traumatic stress. It was pointed out that many of these risk factors have been augmented during the COVID-19 pandemic.

The presentation then turned to reviewing the personal signs of trauma exposure response. Participants were asked to take a moment to assess themselves for any signs of trauma exposure response. A handout was provided that included a checklist of possible signs and symptoms (Appendix C), and participants were encouraged to check which ones applied to them. After a few minutes of reflection, a number of questions were posed to the group to encourage group sharing and discussion.

The presentation continued by defining the concept of trauma stewardship. Trauma stewardship is recognizing the incredible honor as well as the tremendous responsibility of caring for others. Part of trauma stewardship is developing a deep sense of awareness and recognizing the need to care for ourselves while caring for others. Subsequently, clear examples of how to prevent trauma exposure response were reviewed.

To explore the concepts of the PowerPoint presentation more fully, participants were split into small groups, either in person or via virtual breakout groups. If the participants were unable to get into small groups, they were asked to answer the reflection questions on paper individually and then share themes with the larger group. The small-group exercises and reflections are available in Appendix D. The small-group exercises focused on active ways to mitigate trauma exposure response and how to realistically apply this to one's life as a resident or fellow.

After the small-group exercise, participants were encouraged to share a few key discussion points with the larger group. The faculty wellness champion facilitated this and addressed key points.

In closing, the faculty wellness champion reviewed key local resources, including the employee assistance program and other mental health support resources. All participants were instructed to complete the postsession evaluation (Appendix E). This evaluation was modeled after other evaluations used to assess educational activities at our institution.

After all programs completed their sessions, the postsession evaluation results were reviewed. Analysis included basic descriptive statistics. Key words and phrases from the open-ended questions were tagged, and the themes with the highest frequency were identified.

## Results

Between September and December 2020, 17 program wellness sessions were held. One more session was held in February 2021 due to delays from a significant COVID-19 surge. In total, 28 residency and fellowship programs at the institution participated in this program during protected didactic time. The 28 programs included a total of 292 residents and fellows to whom this session was offered. Attendance was not documented but was highly encouraged, and didactic attendance expectations were applied to the session. Fifty-nine participants completed the postsession evaluation.

The postsession evaluation demonstrated that 64% of participants agreed and 25% of participants strongly agreed they had improved their understanding of trauma exposure response due to the session. Sixty-four percent of participants agreed and 34% strongly agreed that after completing this session they understood the signs of trauma exposure response. When asked if after this session they recognized steps that could be taken to cope with trauma exposure response, 57% of participants agreed and 31% of participants strongly agreed. Overall, 15% of trainees rated the session as somewhat valuable, 54% rated it as valuable, and 27% rated it as extremely valuable.

Open-ended questions were also asked on the evaluation. In response to the question “What was most helpful about this session?”, common themes included the following:

• Sincere appreciation for protected time with peers to discuss the topic of trauma exposure response.
• Appreciation for attending physicians sharing their stories and being vulnerable.
• Positive feedback on small-group discussion format.
• The novelty to many trainees of the signs of trauma exposure response as well as the helpfulness of identifying them.

One trainee commented:

Honestly, just taking a moment out of the day to speak about things that mostly wouldn't be discussed was very valuable. I appreciated knowing that it's okay to have certain feelings and learning how to cope with them. Overall, as a junior resident, I really enjoyed the session.

Other trainees noted that the most helpful components of the session were “encouragement, knowing that others have similar experiences,” “addressing signs of burnout and discussing with other residents,” and “focusing on empathy towards yourself to not be so hard on ourselves.”

When asked “What could be improved about this session?”, the most common theme was that an in-person versus virtual setting would be preferred for the discussion. There were also several comments related to desiring more time, particularly to discuss strategies and realistic ways to mitigate trauma exposure response. A number of participants requested additional relevant reading or podcast materials.

## Discussion

This session on trauma exposure response was valued and well received by residents and fellows. It provided a confidential safe space to learn, connect with colleagues, and process unique experiences of medicine.

This session reached most of the trainees at our institution, as it has been an expectation over the last 5 years that programs will protect time for the institution's longitudinal wellness curriculum. Additionally, many faculty have experience in the wellness champion role, are familiar with the necessary facilitation skills, and enjoy the opportunity to weave personal stories and experiences into the session. The institutional and faculty support of this curriculum is a unique strength.

Strengths of the session include the efficient delivery of material, the opportunity for self-reflection, and robust small-group discussion. The session was intentionally developed in the setting of the COVID-19 pandemic and adapted to the unique challenges the pandemic presented. Additionally, the session is portable and able to be presented by a variety of facilitators without extensive background training. The session is short and can be completed in 1 hour, making it easy to implement within existing program structures. No additional funding was required for this session. This session would be applicable to health care professionals outside of residents and fellows.

A limitation of this session is sufficient time for discussion. The session was designed to fit into the typical 1-hour format of didactics, but feedback demonstrated that more time for discussion would have been appreciated on this relevant topic. We recommend considering a duration of 90 minutes for the session. If 90 minutes are available, we suggest allowing more time for reflection and discussion of Appendix C during the PowerPoint presentation as well as more time for large-group discussion and resource review.

Additionally, it is recognized that the solutions to combating trauma exposure response are unique to the individual and can be challenging to apply during the busy lifestyle of residency or fellowship. Offering more practical, concrete solutions to participants would be one way to enhance this session.

We learned that scheduling the sessions months in advance is ideal. There is a degree of administrative burden to assuring each program secures time on the faculty wellness champion schedule during didactic time. This was the main barrier to reaching all programs.

Another lesson learned is the importance of setting ground rules before the start of the session. Each group of trainees has its own unique dynamics. It is important that the facilitator sets and reviews the following ground rules:

• The session is completely confidential.
• The content of the discussion will not be reported, and recording a virtual meeting is prohibited.
• Respectful interactions are expected.
• Sharing is optional; however, what you say may help someone else.
• If a session is virtual, turn on cameras and feel free to use the chat for questions and comments.
• If triggered by any of the topics, feel free to step out and connect to other resources, including the employee assistance program or the faculty wellness champion.

In the past, we have held an orientation for faculty wellness champion facilitators to prepare them to run the sessions. No formal materials exist for the orientation other than reviewing the facilitator guide (Appendix A), PowerPoint (Appendix B), other appendices, and references. In addition to the mechanics of the session, we recommend offering additional skill development around optimal facilitation, including normalizing responses, validating concerns, and identifying those who may be struggling. We also recommend discussing the ground rules listed above.

In the future, the content will be improved based on feedback from trainees and faculty wellness champions. We plan to present this topic again in the coming years as part of the institutional wellness curriculum. We have also adapted this session to use for groups of attending physicians by request. The session also aligns with other wellness programming at our institution (i.e., protected time for debriefing after all critical care rotations for trainees). We continue to work with the faculty and pastoral care teammates who facilitate these monthly debrief sessions since much of the content in the discussions is related to trauma exposure response. It is recognized that due to the COVID-19 pandemic, this topic has only become more relevant and that arming our faculty and trainees with the skills to recognize the signs and know when to reach out for help is critical.

## Appendices


Facilitator Guide.docxTrauma Exposure Response Presentation.pptxTrauma Exposure Response Handout.docxSmall-Group Exercises and Reflection Questions.docxPostsession Evaluation.docx
*All appendices are peer reviewed as integral parts of the Original Publication.*

## References

[1] CLER Evaluation Committee. CLER Pathways to Excellence: Expectations for an Optimal Clinical Learning Environment to Achieve Safe and High-Quality Patient Care, Version 2.0. Accreditation Council for Graduate Medical Education; 2019. Accessed September 7, 2021. https://www.acgme.org/Portals/0/PDFs/CLER/1079ACGME-CLER2019PTE-BrochDigital.pdf

[2] Accreditation Council for Graduate Medical Education. ACGME Common Program Requirements (Residency). Accreditation Council for Graduate Medical Education; 2021. Accessed September 7, 2021. https://www.acgme.org/Portals/0/PFAssets/ProgramRequirements/CPRResidency2021.pdf

[3] Accreditation Council for Graduate Medical Education. ACGME Common Program Requirements Section VI With Background and Intent. Accreditation Council for Graduate Medical Education; 2017. Accessed September 7, 2021. https://www.acgme.org/Portals/0/PFAssets/ProgramRequirements/CPRs_Section%20VI_with-Background-and-Intent_2017-01.pdf

[4] Shanafelt TD, Noseworthy JH. Executive leadership and physician well-being: nine organizational strategies to promote engagement and reduce burnout. Mayo Clin Proc. 2017;92(1):129–146. 10.1016/j.mayocp.2016.10.004 27871627

[5] Panagioti M, Panagopoulou E, Bower P, et al. Controlled interventions to reduce burnout in physicians: a systemic review and meta-analysis. JAMA Intern Med. 2017;177(2):195–205. 10.1001/jamainternmed.2016.7674 27918798

[6] Shanafelt TD, West CP, Sinsky C, et al. Changes in burnout and satisfaction with work-life integration in physicians and the general US working population between 2011 and 2017. Mayo Clin Proc. 2019;94(9):1681–1694. 10.1016/j.mayocp.2018.10.023 30803733

[7] Cieslak R, Shoji K, Douglas A, Melville E, Luszczynska A, Benight CC. A meta-analysis of the relationship between job burnout and secondary traumatic stress among workers with indirect exposure to trauma. Psychol Serv. 2014;11(1):75–86. 10.1037/a0033798 23937082

[8] Shoji K, Lesnierowska M, Smoktunowicz E, et al. What comes first, job burnout or secondary traumatic stress? Findings from two longitudinal studies from the U.S. and Poland. PLoS One. 2015;10(8):e0136730. 10.1371/journal.pone.0136730 26305222 PMC4549333

[9] Tyrie L, Mosenthal A, Bryczkowski S, Laboy C, Lamba S. Difficult conversations after resuscitation in trauma: video education e-module. MedEdPORTAL. 2015;11:10092. 10.15766/mep_2374-8265.10092

[10] Martinchek M, Bird A, Pincavage AT. Building team resilience and debriefing after difficult clinical events: a resilience curriculum for team leaders. MedEdPORTAL. 2017;13:10601. 10.15766/mep_2374-8265.10601 30800803 PMC6338182

[11] Van Dernoot Lipsky L. Trauma Stewardship: An Everyday Guide to Caring for Self While Caring for Others. Berrett-Koehler Publishers; 2009.

