# Supplementary material for: Trauma Exposure Response: How Secondary Trauma Affects Personal and Professional Life
Source: MedEdPORTAL. 2021 Nov 22;17:11192. doi: 10.15766/mep_2374-8265.11192 (PMC8607743; doi:10.15766/mep_2374-8265.11192)
Supplement: Supplementary file 1 — Facilitator Guide.docxTrauma Exposure Response Presentation.pptxTrauma Exposure Response Handout.docxSmall-Group Exercises and Reflection Questions.docxPostsession Evaluation.docx [file mep_2374-8265.11192-s001.zip › A. Facilitator Guide.docx]

Trauma Exposure Response

Facilitator Guide

Thank you for participating in this session! Below are recommendations regarding the structure of this session. The faculty champion is welcome to revise/adapt this program slightly if necessary. Personal anecdotes and experience are encouraged to be shared.

Please contact _______ with any questions

**Learning Objectives:** By participating in the resident wellness curricula, the resident will be able to:

1. Define the concepts of secondary trauma and trauma exposure response

2. Recognize the signs of trauma exposure response

3. Demonstrate techniques that can be applied to cope with trauma exposure response

Each program has been asked to identify a “Wellness Champion.” This may be a physician faculty member or non-physician leader as seen fit by the program director. Employee assistance program clinicians are also available to be present and help with the session if desired by the program.

**The responsibilities of the Wellness Champion will include:**

- Delivering the PowerPoint presentation
- Organizing and facilitating the small group discussion
- Encourage participants to complete the post-session evaluation at the end of the presentation.

**The responsibilities of the Program Coordinator will include:**

- Schedule program wellness session
- Print handouts if in person session
- Consider setting up a zoom session to use breakout groups for larger, virtual groups.

# Trauma Exposure Response Presenter guide

**It is recognized that due to COVID19 many wellness sessions will be virtual instead of in person. If there is capability to use Zoom breakout rooms for small group discussion, this would be ideal. If not possible, participants are encouraged to reflect on the discussion questions individually, write down reflections, and then share themes with the larger group.

# Introduction and Gratefulness (5 min)

Each year our institution directs formal wellness curriculum for our participants. This year the topic is Trauma Exposure Response. Secondary trauma is the emotional distress that results when an individual hears about the firsthand trauma experiences of another. Physicians are at risk for secondary trauma especially during the COVID19 pandemic. During this pandemic, health-care workers are facing traumatic experiences in both their professional and personal worlds.

*If you have a smaller group, we suggest starting the session with each participant sharing something they are grateful for, or sharing something positive about their life outside of work

# Power Point Presentation: (15min)

## The Power Point will be sent to the faculty wellness Champion ahead of time to review. Feel free to use suggested text in the notes section or supplement with your own expertise.

- - Of note, at slide 9 of the PowerPoint presentation it is recommended to pause for 5 minutes for the participants to reflect and assess for any signs of trauma exposure response (Appendix C).

# Small Group Discussion (25 minutes)

## Group Discussion Format

### Break up into small groups (Recommend 4-6 participants per group-dependent on size of overall group). If possible, to do by zoom breakout rooms for virtual sessions, this would be ideal. If not, ask participants to reflect for a few minutes on the questions and write down their reflections.

### Distribute the discussion questions and have teams discuss as a small group.

### Report out (10 minutes)

1. Ask groups to share a few key discussion points from their discussion with the large group.
   1. If participants did individual reflections, invite participants to share with the larger group or call on specific participants to share if comfortable.
2. **Closing: (5 minutes)**

## Point out the resources to know on the last slide.

1. Remind participants to complete the post-session evaluation.
