# Supplementary material for: Trauma Exposure Response: How Secondary Trauma Affects Personal and Professional Life
Source: MedEdPORTAL. 2021 Nov 22;17:11192. doi: 10.15766/mep_2374-8265.11192 (PMC8607743; doi:10.15766/mep_2374-8265.11192)
Supplement: Supplementary file 1 — Facilitator Guide.docxTrauma Exposure Response Presentation.pptxTrauma Exposure Response Handout.docxSmall-Group Exercises and Reflection Questions.docxPostsession Evaluation.docx [file mep_2374-8265.11192-s001.zip › B. Trauma Exposure Response Presentation.pptx]

## Slide 1
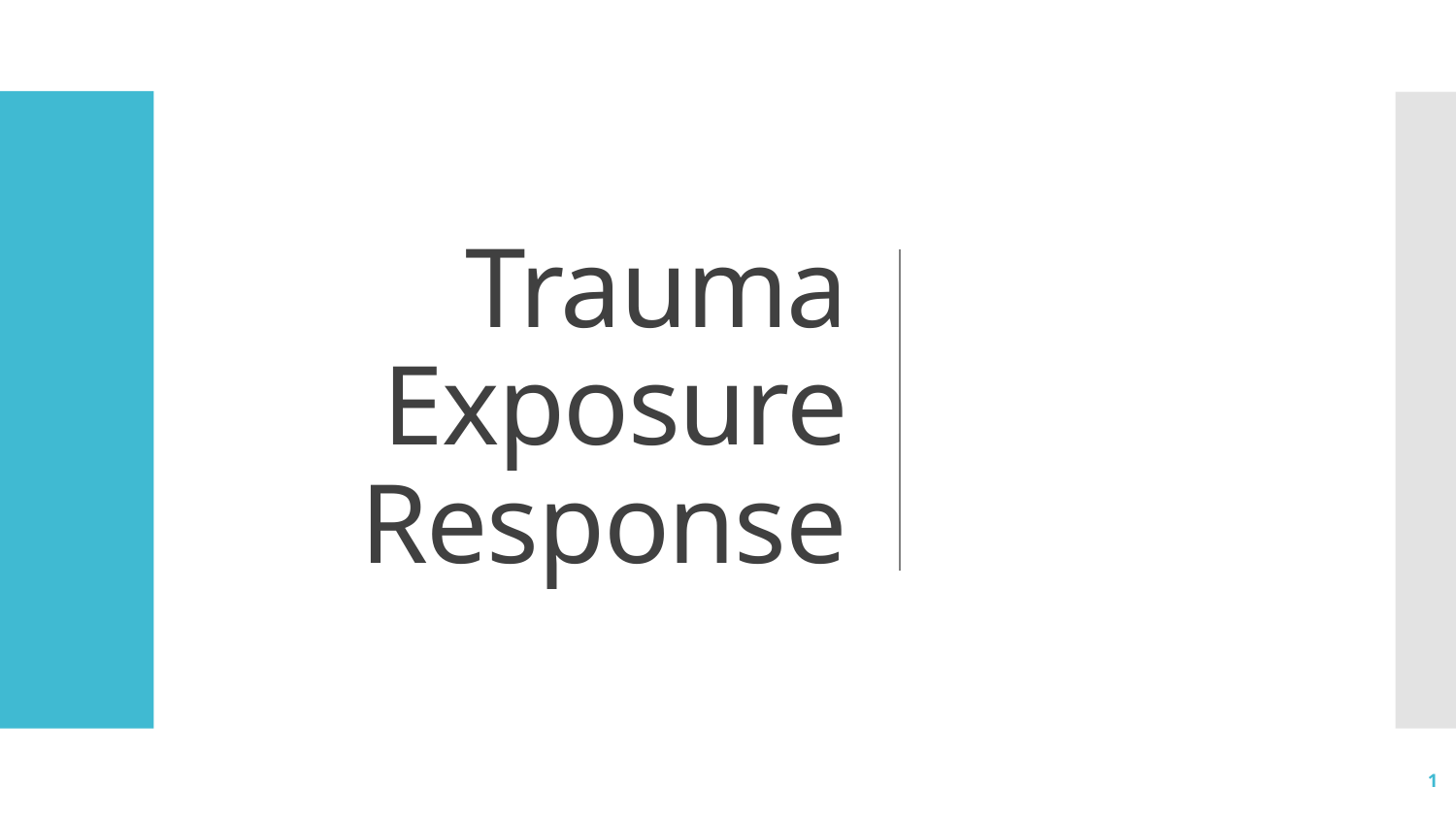

# Trauma Exposure Response
1

## Slide 2
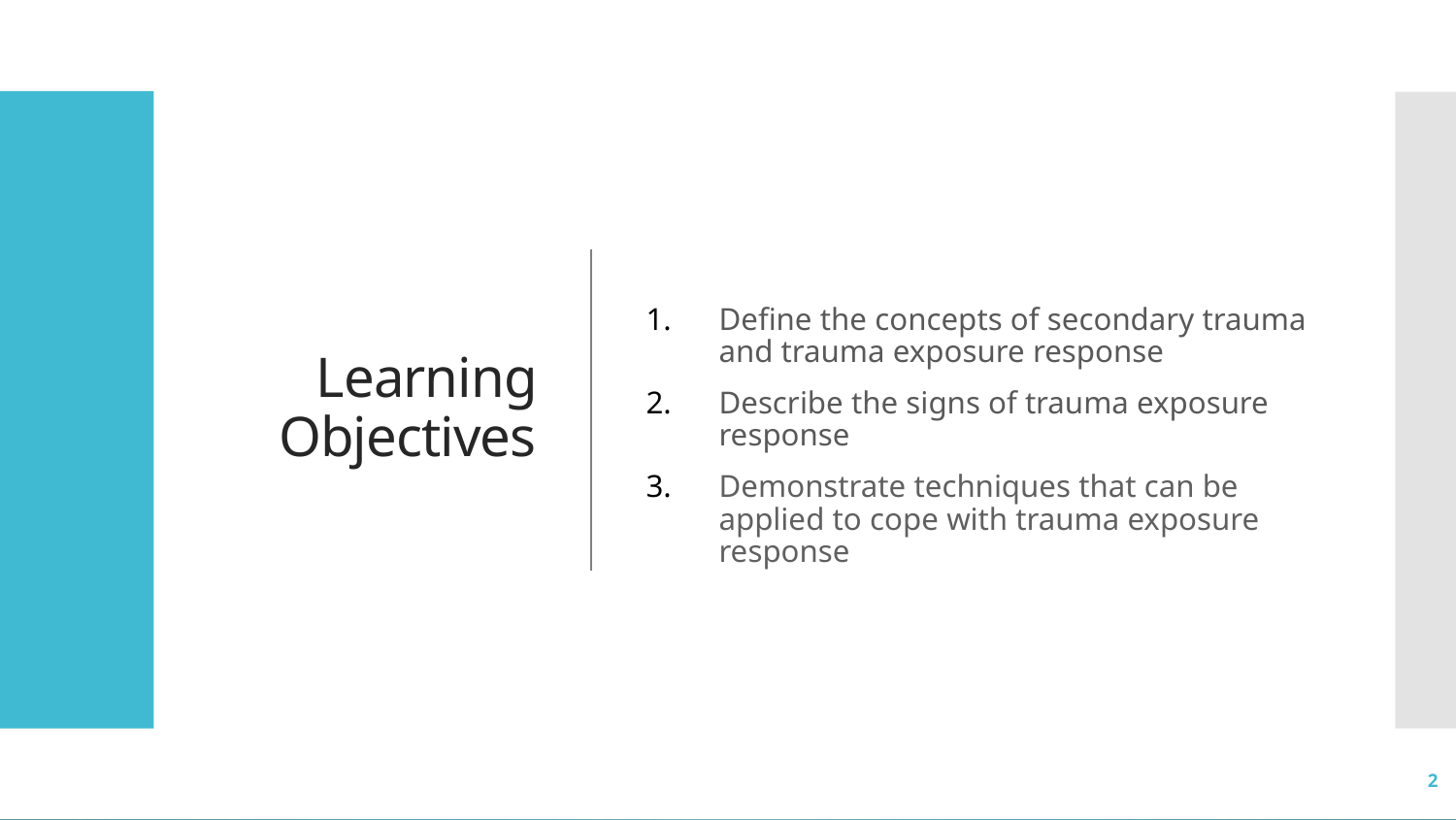

# Learning Objectives
Define the concepts of secondary trauma and trauma exposure response
Describe the signs of trauma exposure response
Demonstrate techniques that can be applied to cope with trauma exposure response
2

## Slide 3
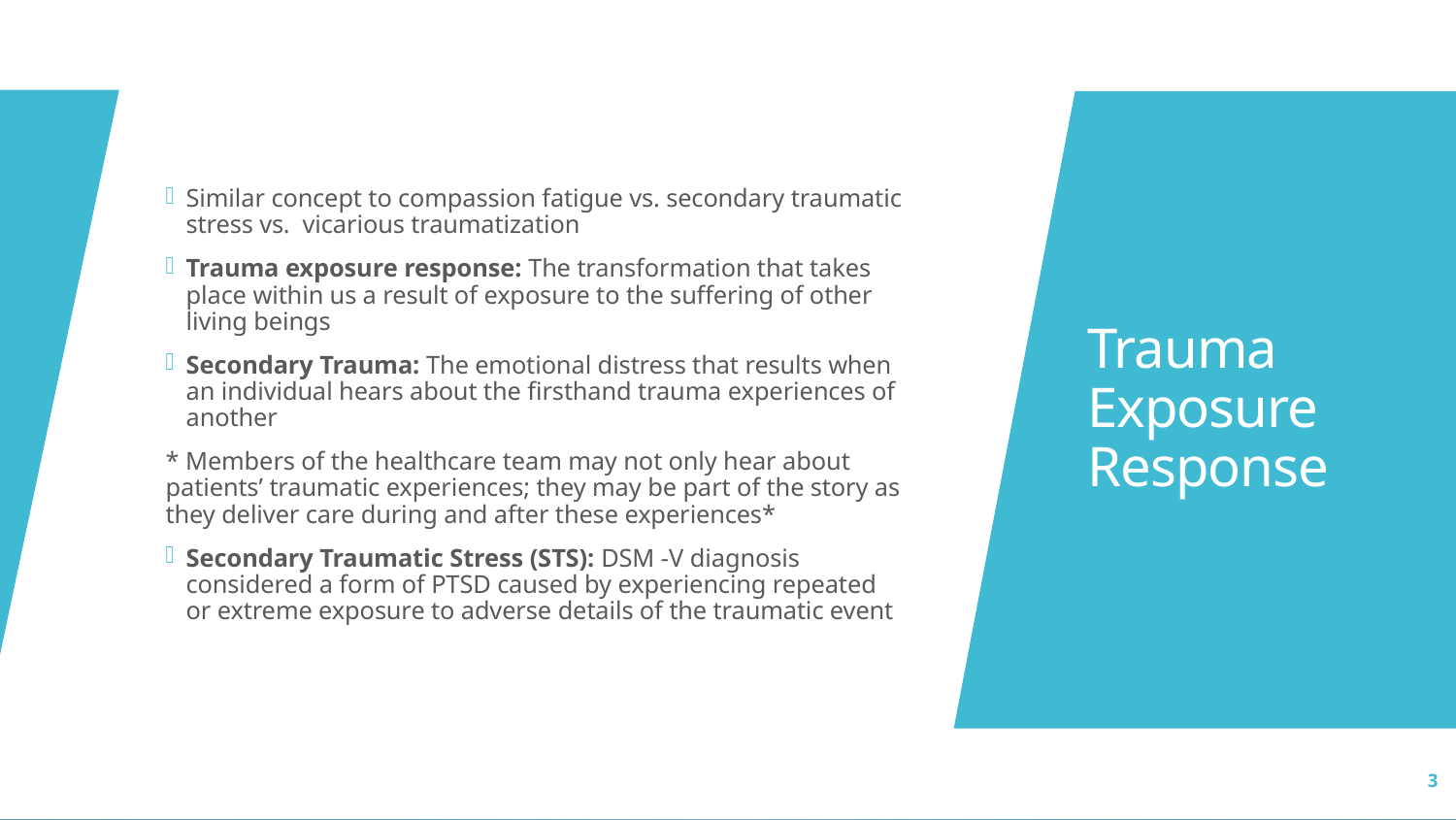

Similar concept to compassion fatigue vs. secondary traumatic stress vs. vicarious traumatization
Trauma exposure response: The transformation that takes place within us a result of exposure to the suffering of other living beings
Secondary Trauma: The emotional distress that results when an individual hears about the firsthand trauma experiences of another
* Members of the healthcare team may not only hear about patients’ traumatic experiences; they may be part of the story as they deliver care during and after these experiences*
Secondary Traumatic Stress (STS): DSM -V diagnosis considered a form of PTSD caused by experiencing repeated or extreme exposure to adverse details of the traumatic event
# Trauma Exposure Response
3

## Slide 4
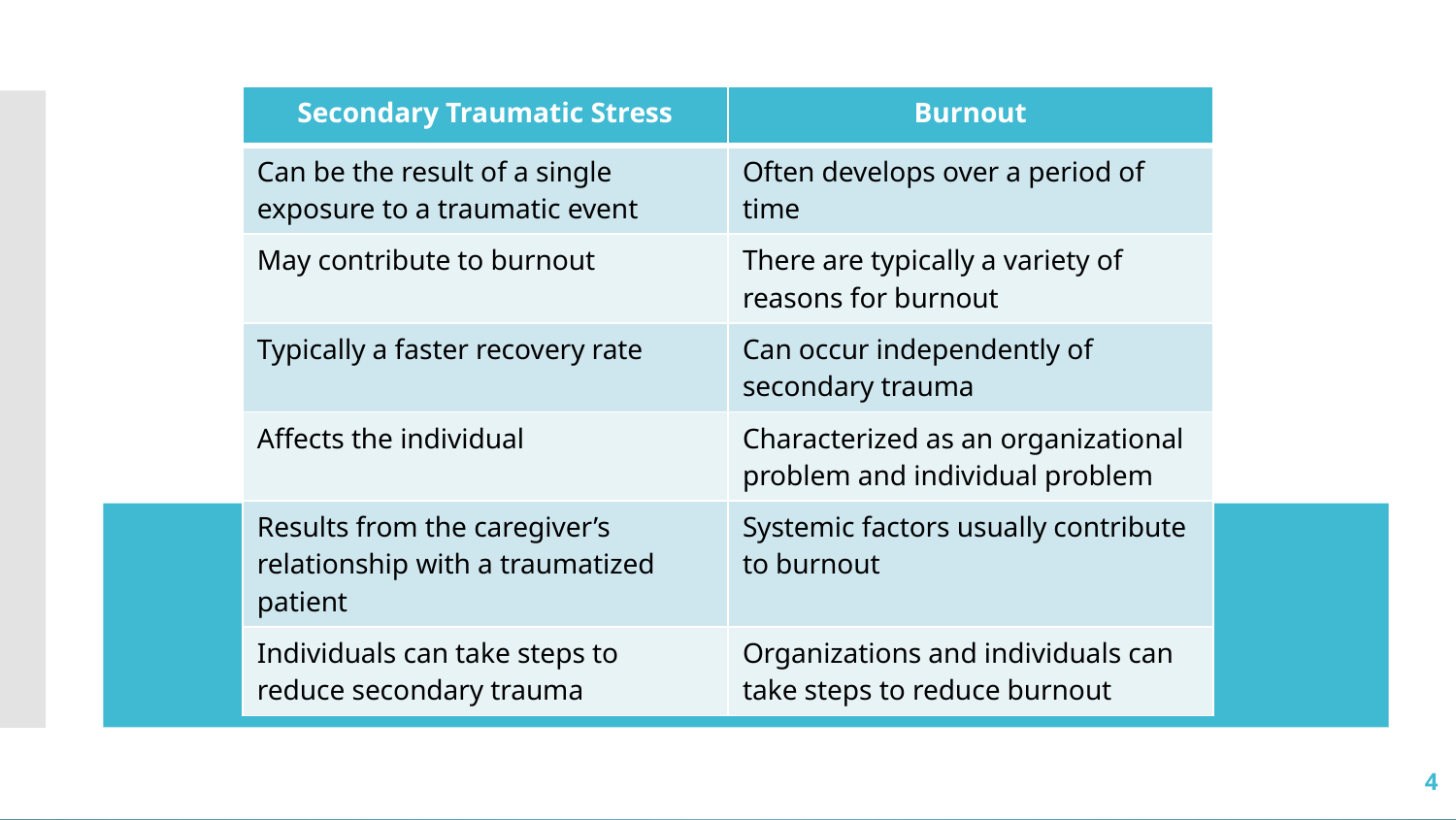

| Secondary Traumatic Stress | Burnout |
| --- | --- |
| Can be the result of a single exposure to a traumatic event | Often develops over a period of time |
| May contribute to burnout | There are typically a variety of reasons for burnout |
| Typically a faster recovery rate | Can occur independently of secondary trauma |
| Affects the individual | Characterized as an organizational problem and individual problem |
| Results from the caregiver’s relationship with a traumatized patient | Systemic factors usually contribute to burnout |
| Individuals can take steps to reduce secondary trauma | Organizations and individuals can take steps to reduce burnout |
4

## Slide 5
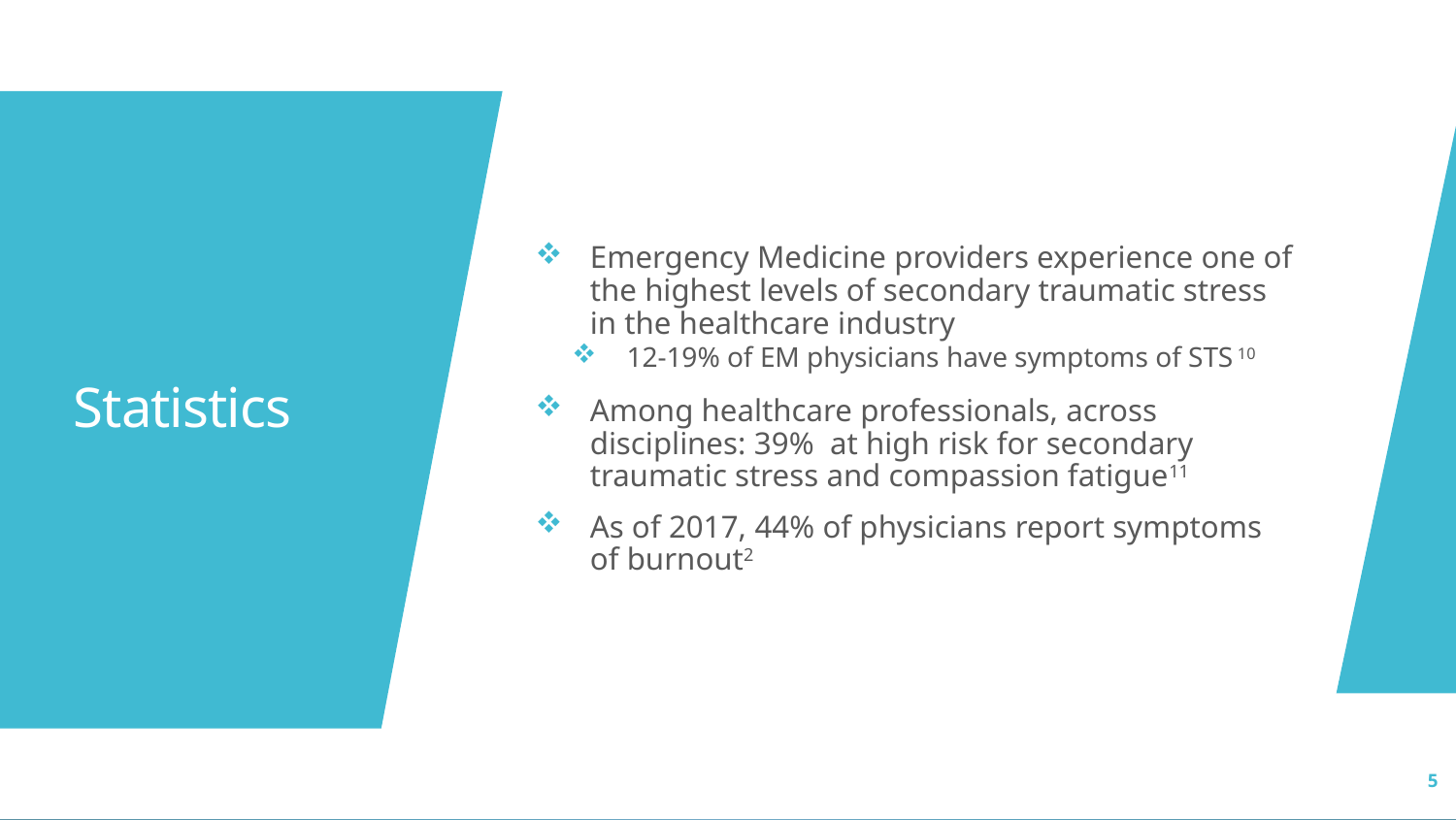

# Statistics
Emergency Medicine providers experience one of the highest levels of secondary traumatic stress in the healthcare industry
12-19% of EM physicians have symptoms of STS 10
Among healthcare professionals, across disciplines: 39% at high risk for secondary traumatic stress and compassion fatigue11
As of 2017, 44% of physicians report symptoms of burnout2
5

## Slide 6
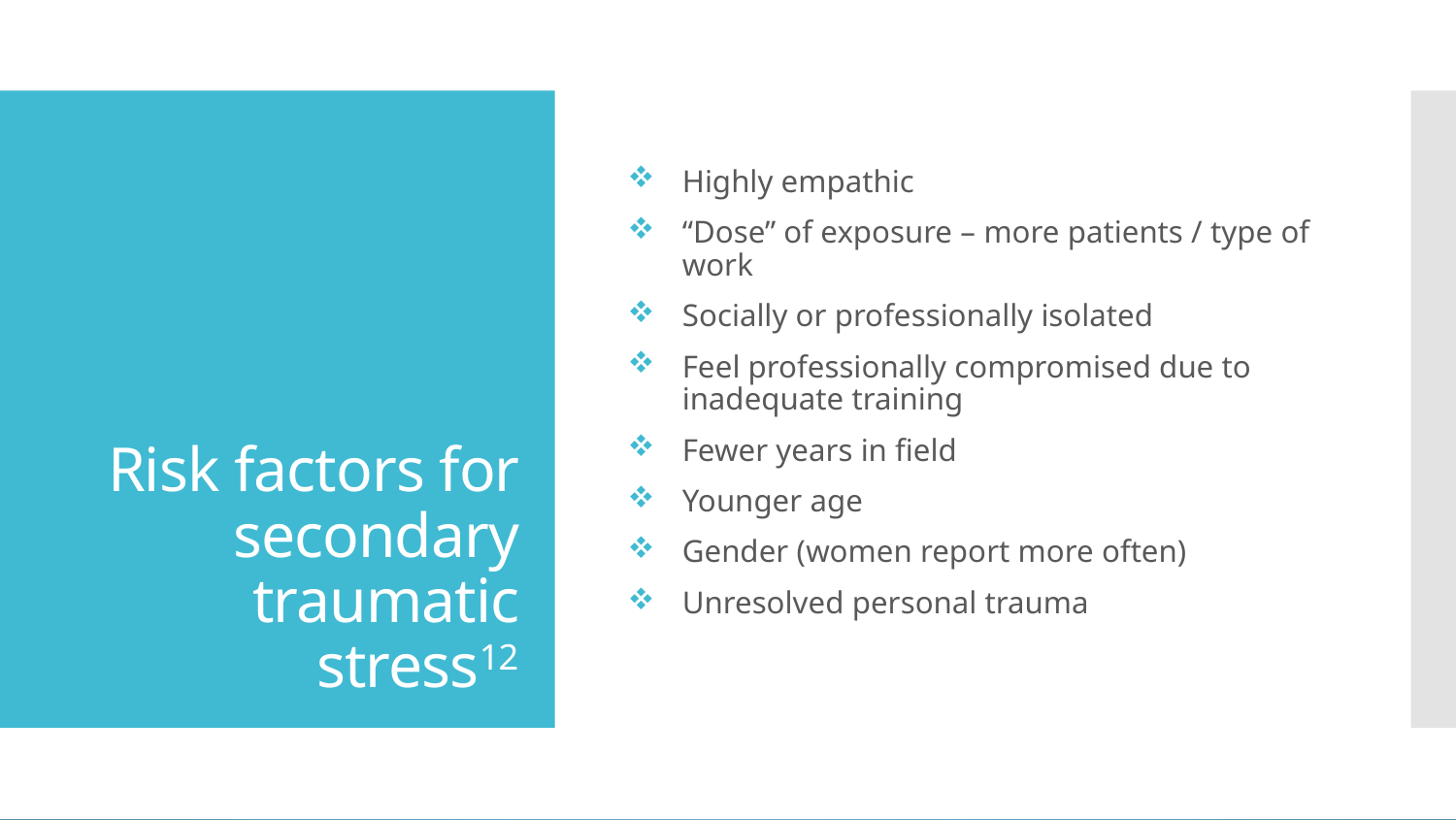

# Risk factors for secondary traumatic stress12
Highly empathic
“Dose” of exposure – more patients / type of work
Socially or professionally isolated
Feel professionally compromised due to inadequate training
Fewer years in field
Younger age
Gender (women report more often)
Unresolved personal trauma

## Slide 7
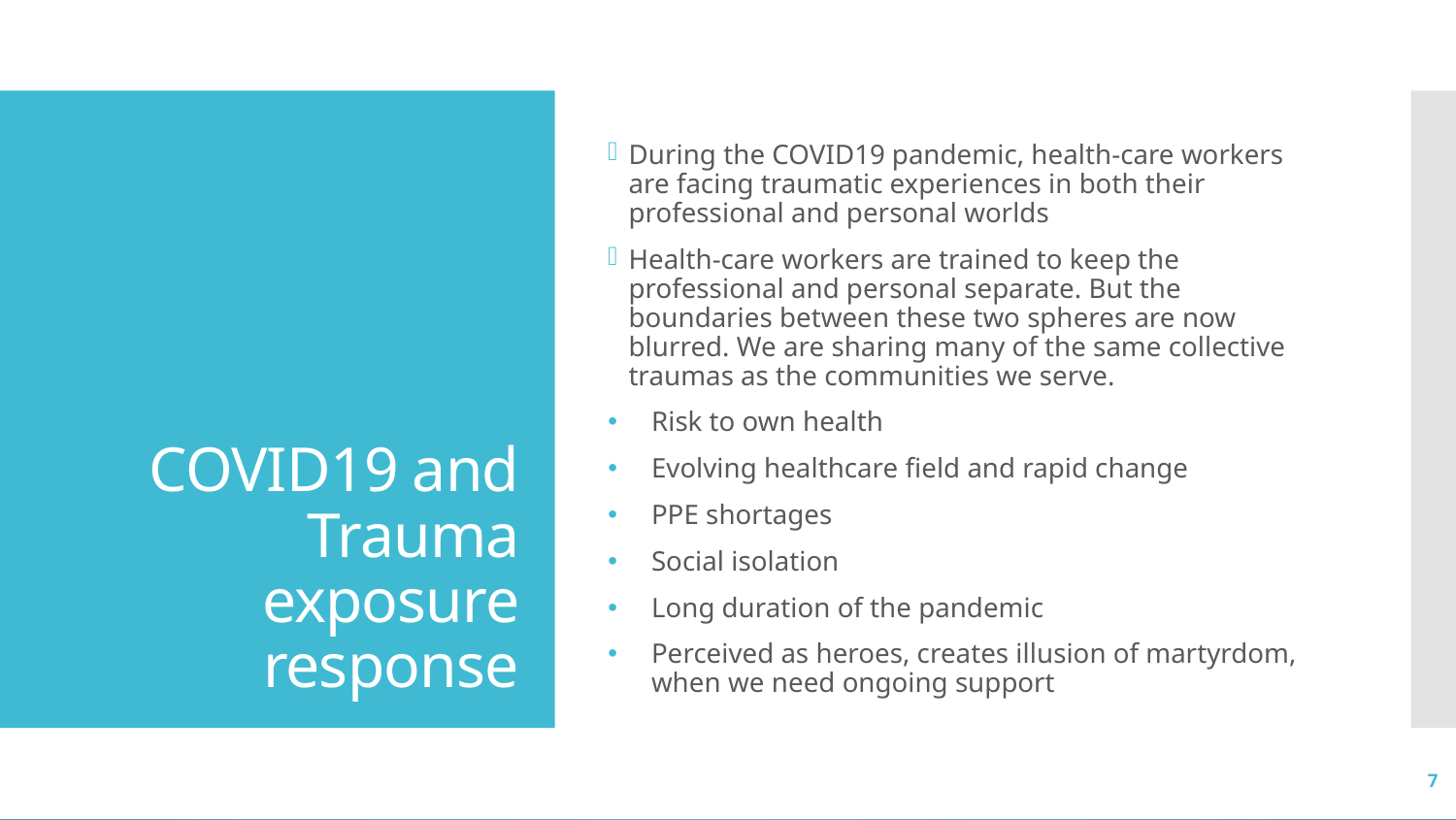

During the COVID19 pandemic, health-care workers are facing traumatic experiences in both their professional and personal worlds
Health-care workers are trained to keep the professional and personal separate. But the boundaries between these two spheres are now blurred. We are sharing many of the same collective traumas as the communities we serve.
Risk to own health
Evolving healthcare field and rapid change
PPE shortages
Social isolation
Long duration of the pandemic
Perceived as heroes, creates illusion of martyrdom, when we need ongoing support
# COVID19 and Trauma exposure response
7

## Slide 8
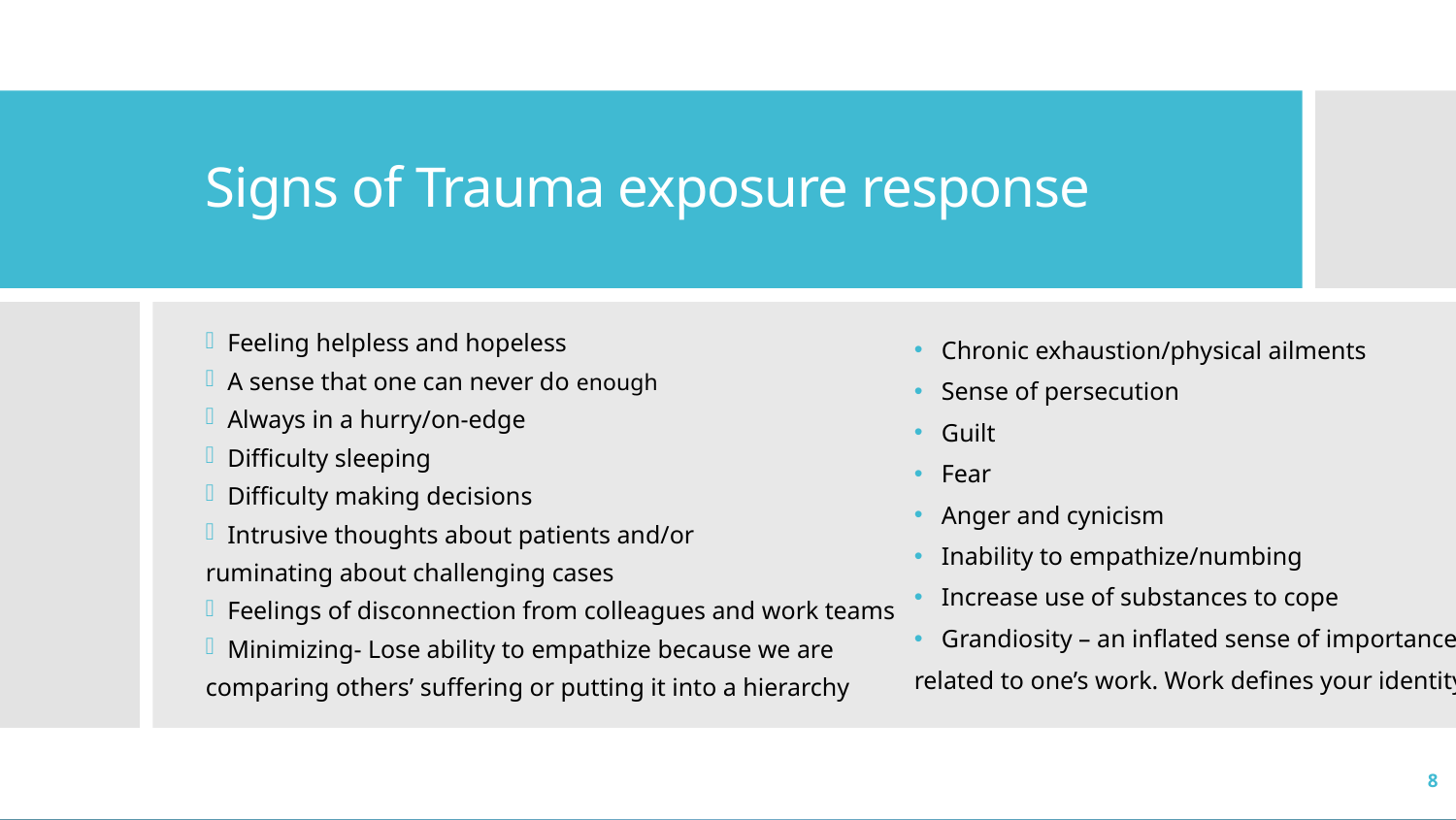

# Signs of Trauma exposure response
Feeling helpless and hopeless
A sense that one can never do enough
Always in a hurry/on-edge
Difficulty sleeping
Difficulty making decisions
Intrusive thoughts about patients and/or
ruminating about challenging cases
Feelings of disconnection from colleagues and work teams
Minimizing- Lose ability to empathize because we are
comparing others’ suffering or putting it into a hierarchy
Chronic exhaustion/physical ailments
Sense of persecution
Guilt
Fear
Anger and cynicism
Inability to empathize/numbing
Increase use of substances to cope
Grandiosity – an inflated sense of importance
related to one’s work. Work defines your identity
8

## Slide 9
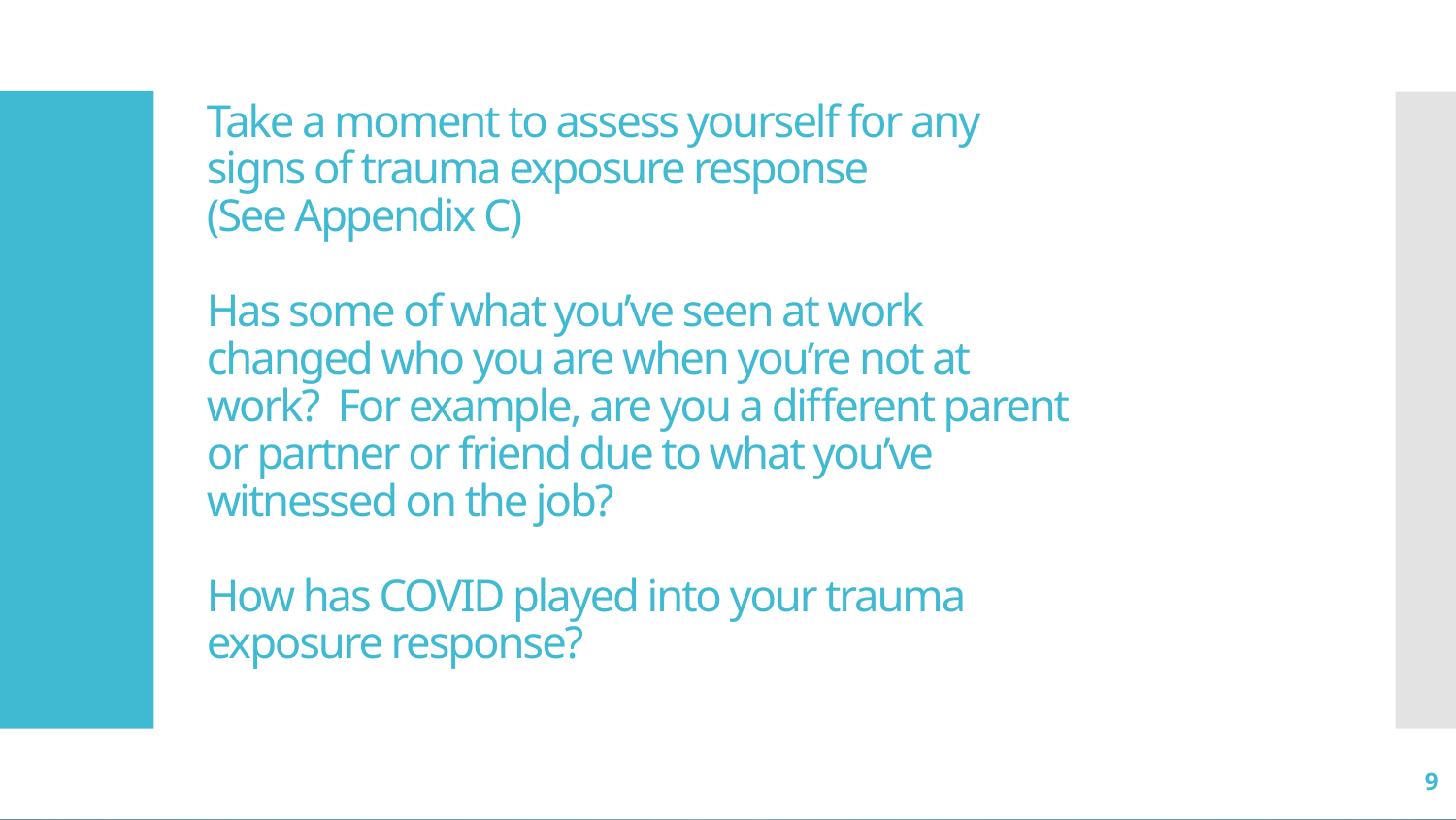

# Take a moment to assess yourself for any signs of trauma exposure response (See Appendix C) Has some of what you’ve seen at work changed who you are when you’re not at work? For example, are you a different parent or partner or friend due to what you’ve witnessed on the job? How has COVID played into your trauma exposure response?
9

## Slide 10
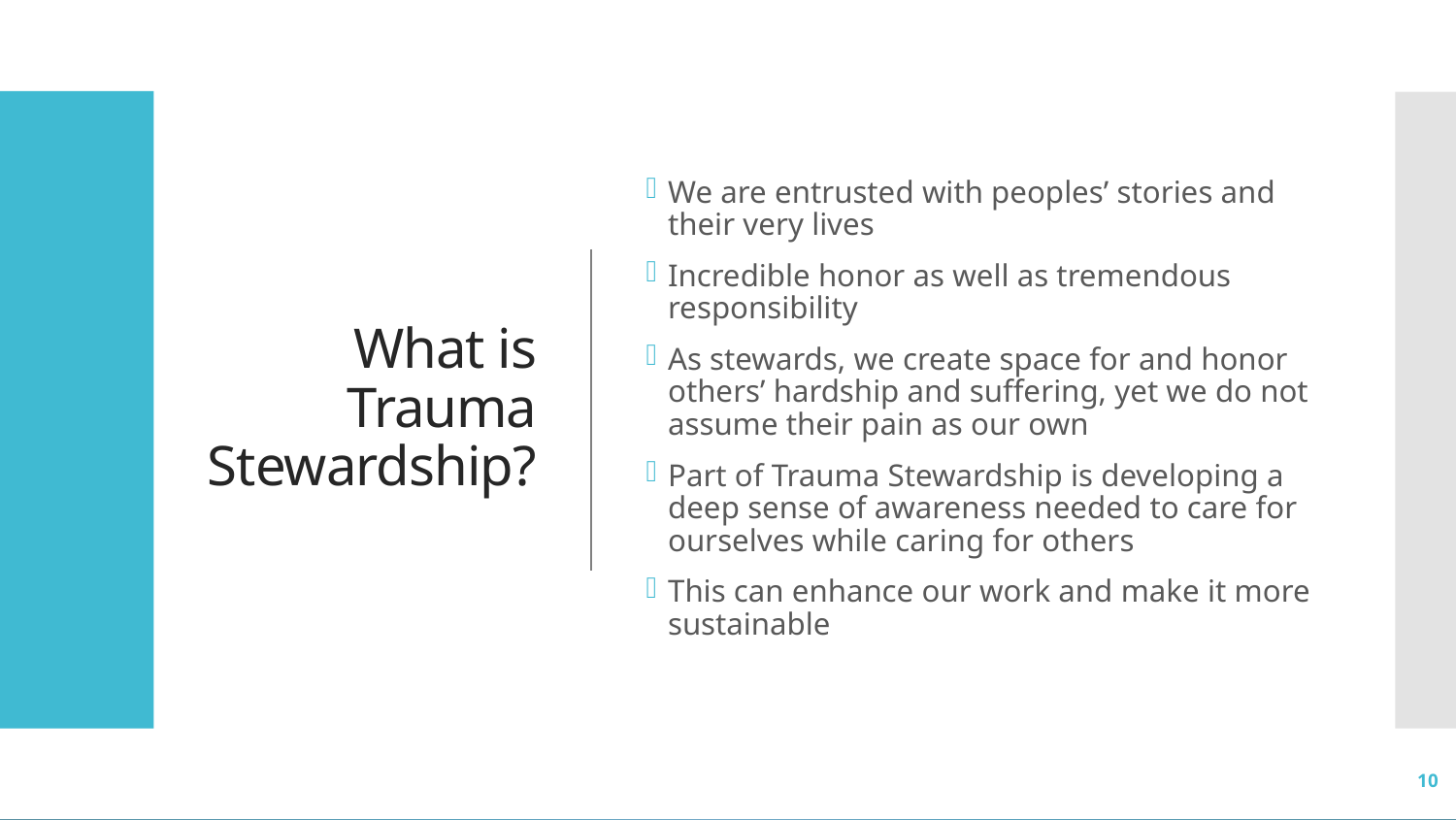

# What is Trauma Stewardship?
We are entrusted with peoples’ stories and their very lives
Incredible honor as well as tremendous responsibility
As stewards, we create space for and honor others’ hardship and suffering, yet we do not assume their pain as our own
Part of Trauma Stewardship is developing a deep sense of awareness needed to care for ourselves while caring for others
This can enhance our work and make it more sustainable
10

## Slide 11
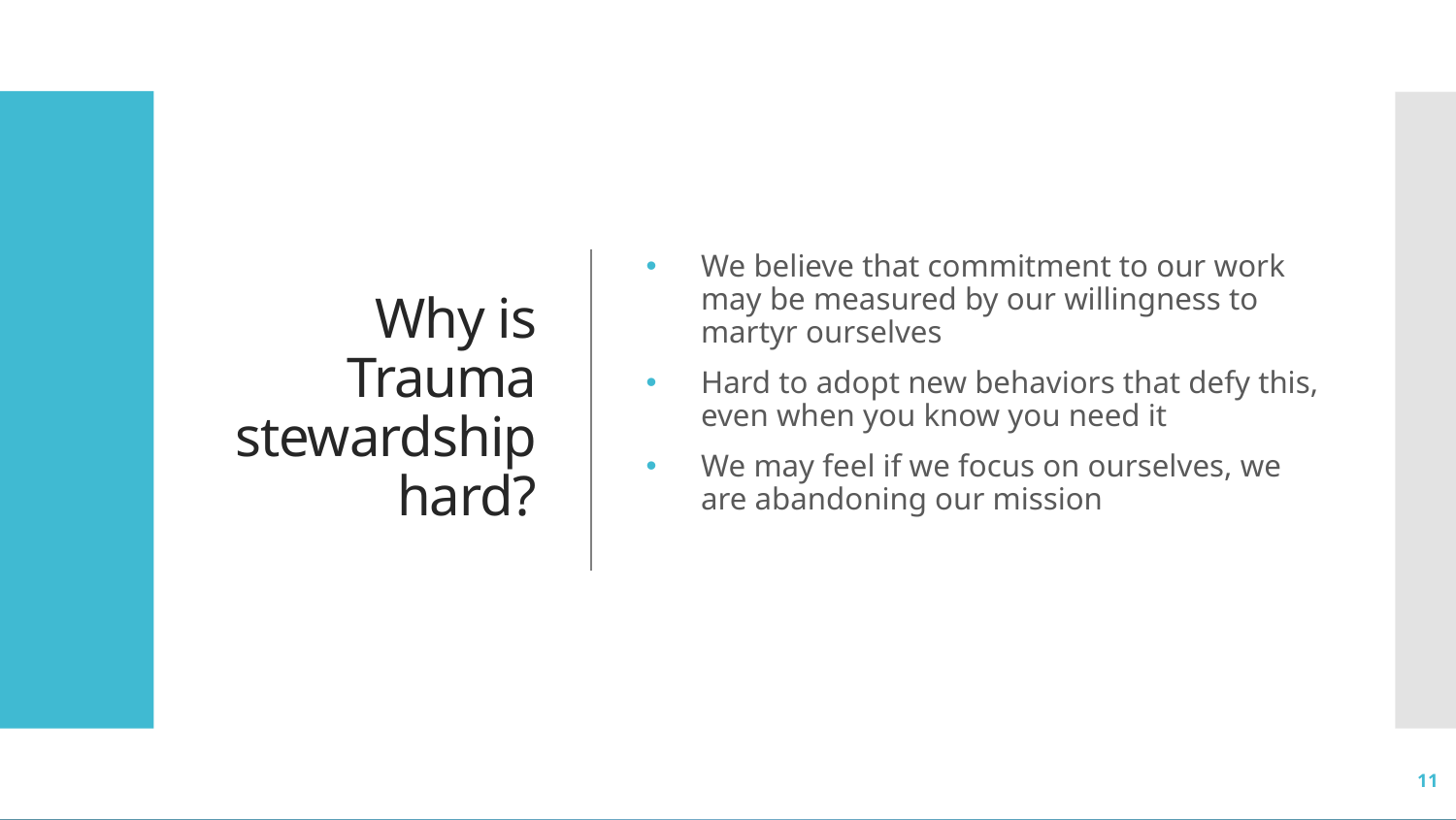

# Why is Trauma stewardship hard?
We believe that commitment to our work may be measured by our willingness to martyr ourselves
Hard to adopt new behaviors that defy this, even when you know you need it
We may feel if we focus on ourselves, we are abandoning our mission
11

## Slide 12
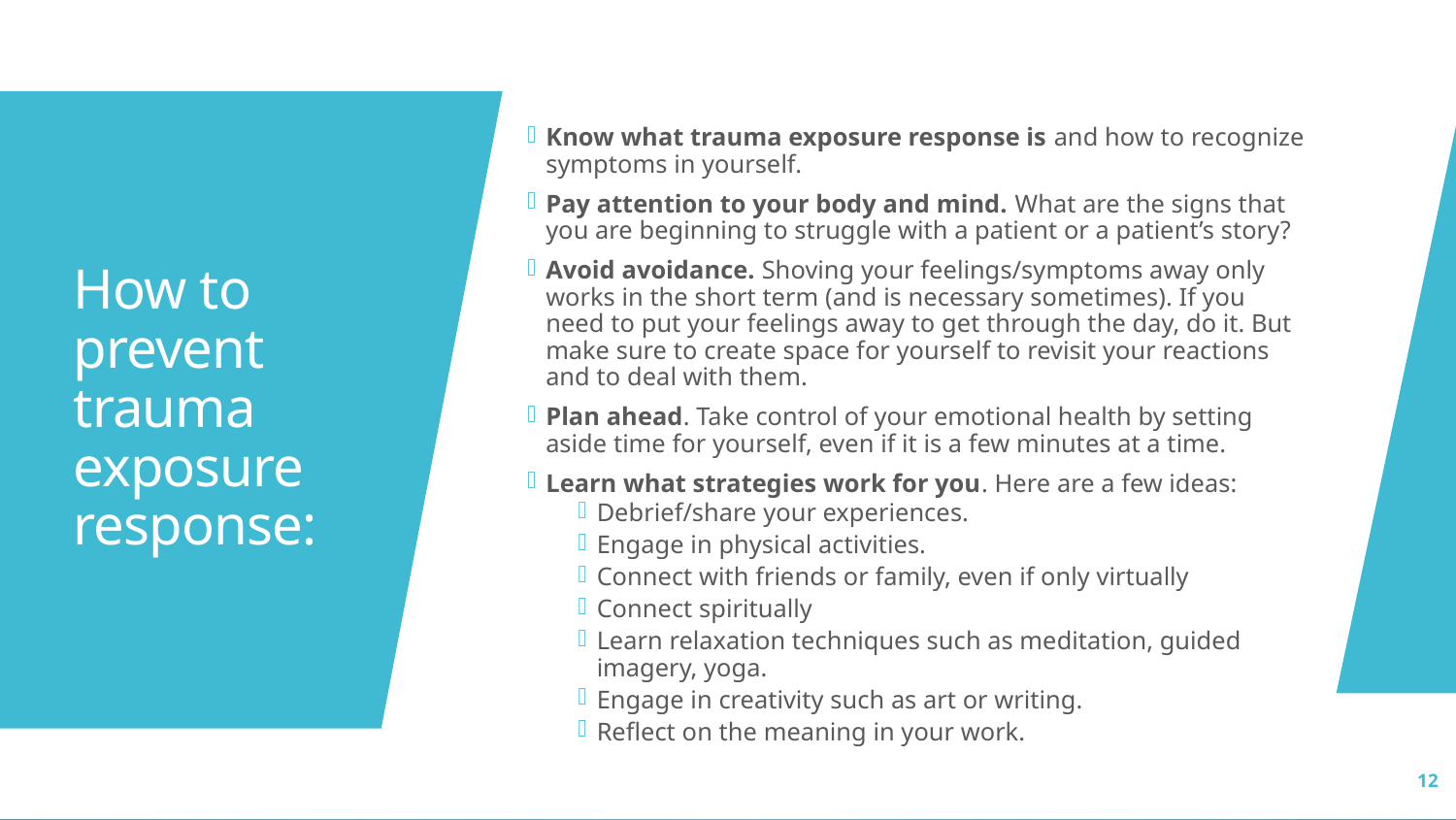

Know what trauma exposure response is and how to recognize symptoms in yourself.
Pay attention to your body and mind. What are the signs that you are beginning to struggle with a patient or a patient’s story?
Avoid avoidance. Shoving your feelings/symptoms away only works in the short term (and is necessary sometimes). If you need to put your feelings away to get through the day, do it. But make sure to create space for yourself to revisit your reactions and to deal with them.
Plan ahead. Take control of your emotional health by setting aside time for yourself, even if it is a few minutes at a time.
Learn what strategies work for you. Here are a few ideas:
Debrief/share your experiences.
Engage in physical activities.
Connect with friends or family, even if only virtually
Connect spiritually
Learn relaxation techniques such as meditation, guided imagery, yoga.
Engage in creativity such as art or writing.
Reflect on the meaning in your work.
# How to prevent trauma exposure response:
12

## Slide 13
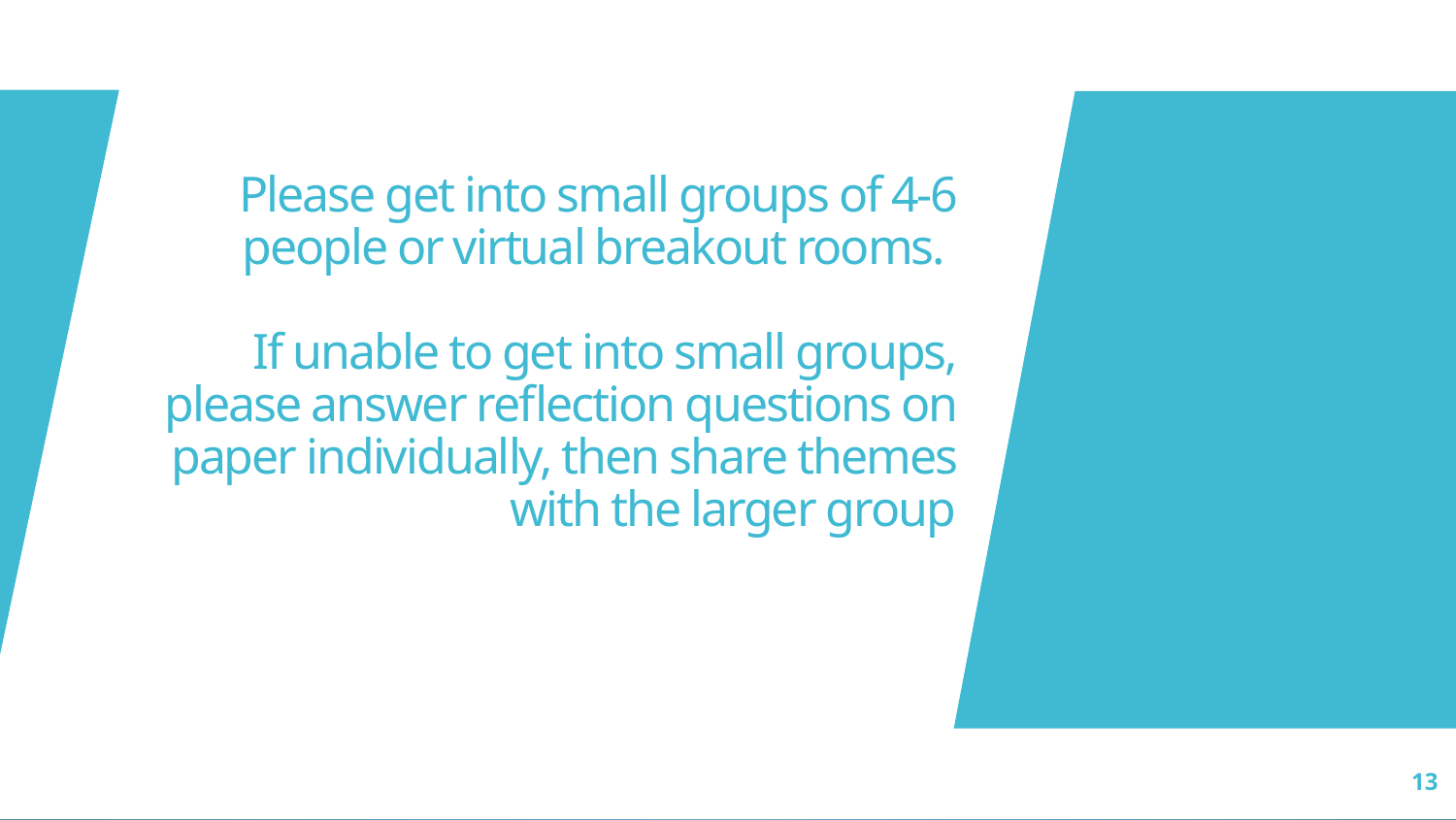

# Please get into small groups of 4-6 people or virtual breakout rooms. If unable to get into small groups, please answer reflection questions on paper individually, then share themes with the larger group
13

## Slide 14
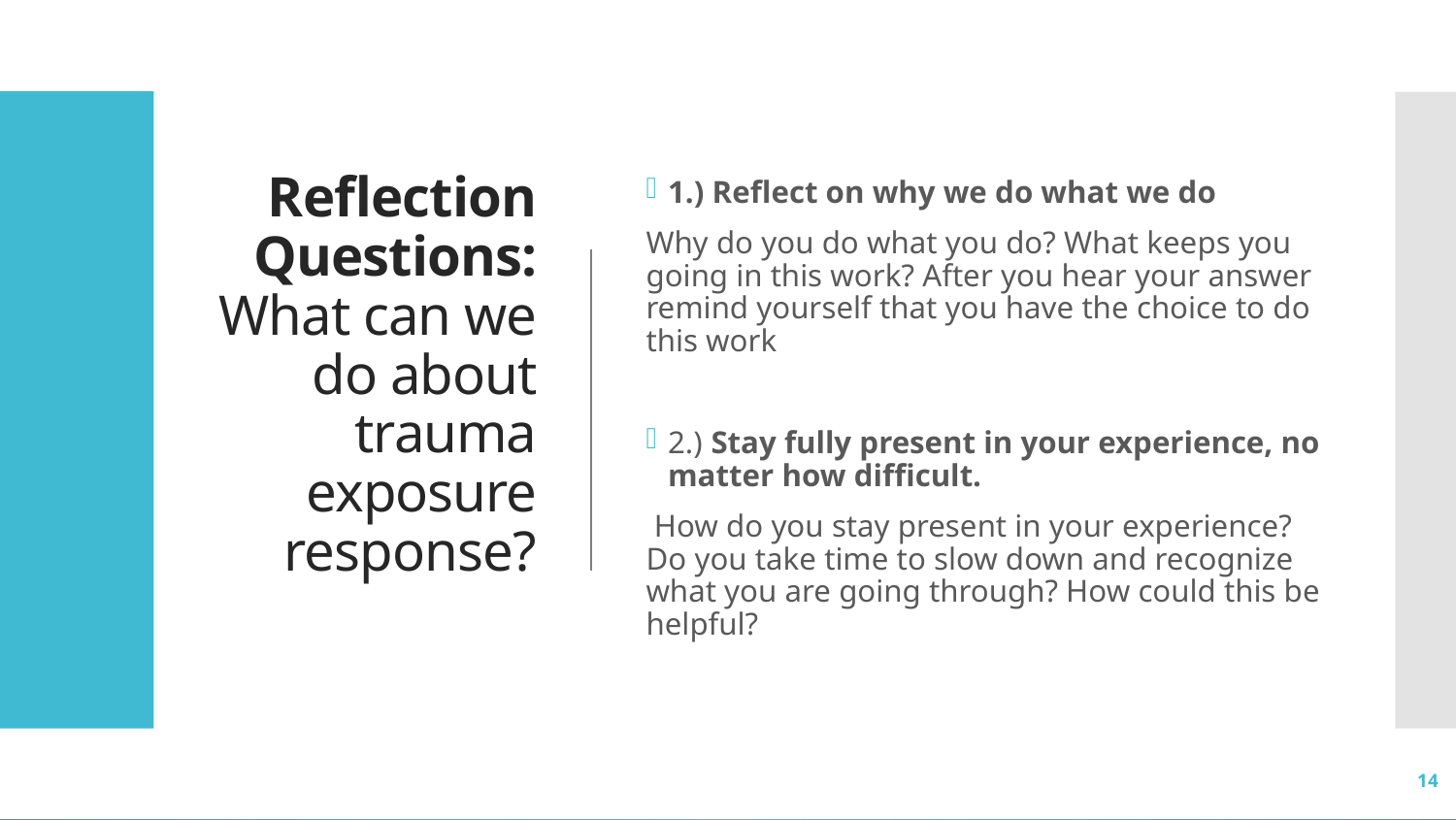

# Reflection Questions: What can we do about trauma exposure response?
1.) Reflect on why we do what we do
Why do you do what you do? What keeps you going in this work? After you hear your answer remind yourself that you have the choice to do this work
2.) Stay fully present in your experience, no matter how difficult.
 How do you stay present in your experience? Do you take time to slow down and recognize what you are going through? How could this be helpful?
14

## Slide 15
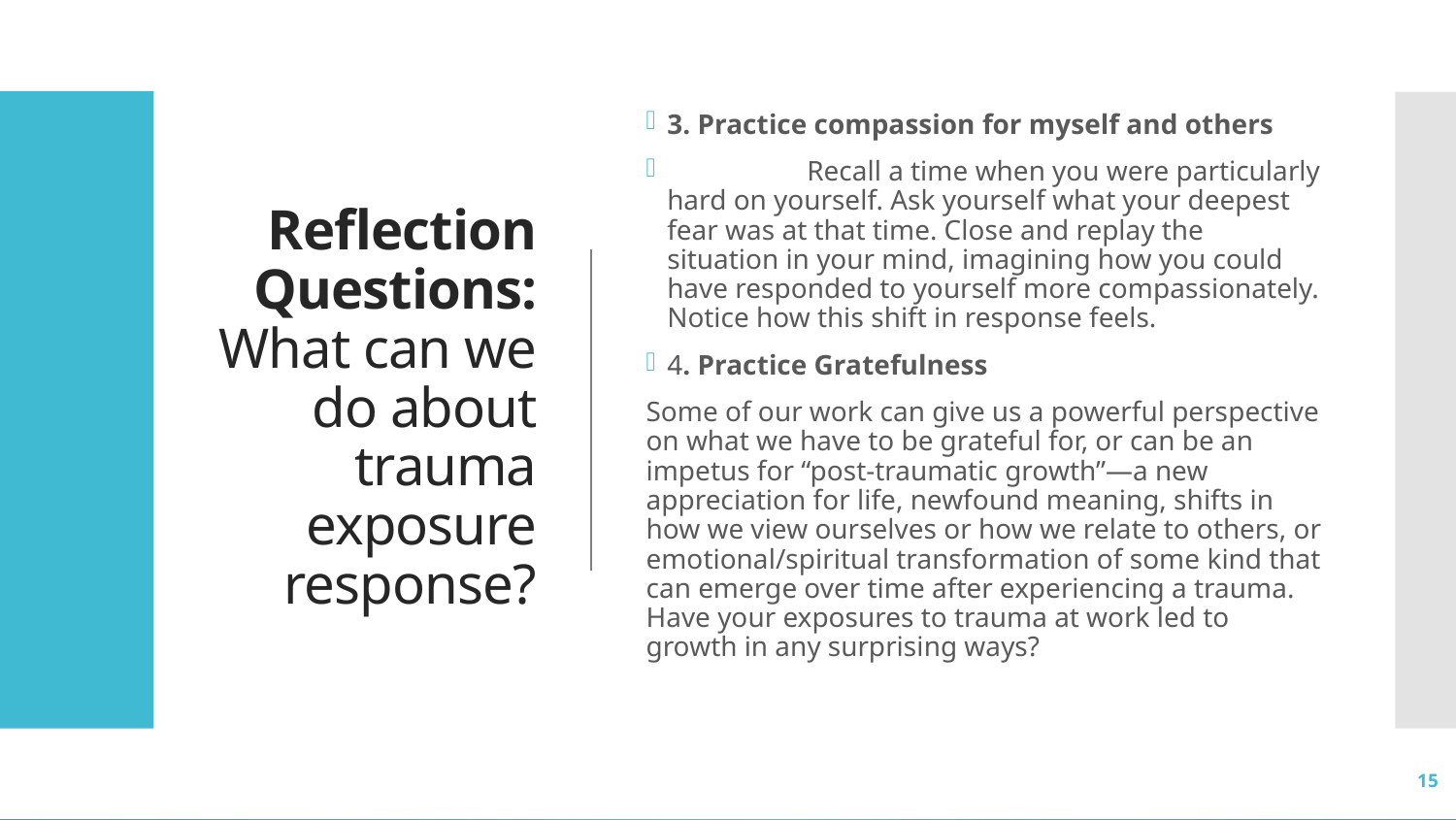

# Reflection Questions: What can we do about trauma exposure response?
3. Practice compassion for myself and others
	Recall a time when you were particularly hard on yourself. Ask yourself what your deepest fear was at that time. Close and replay the situation in your mind, imagining how you could have responded to yourself more compassionately. Notice how this shift in response feels.
4. Practice Gratefulness
Some of our work can give us a powerful perspective on what we have to be grateful for, or can be an impetus for “post-traumatic growth”—a new appreciation for life, newfound meaning, shifts in how we view ourselves or how we relate to others, or emotional/spiritual transformation of some kind that can emerge over time after experiencing a trauma. Have your exposures to trauma at work led to growth in any surprising ways?
15

## Slide 16
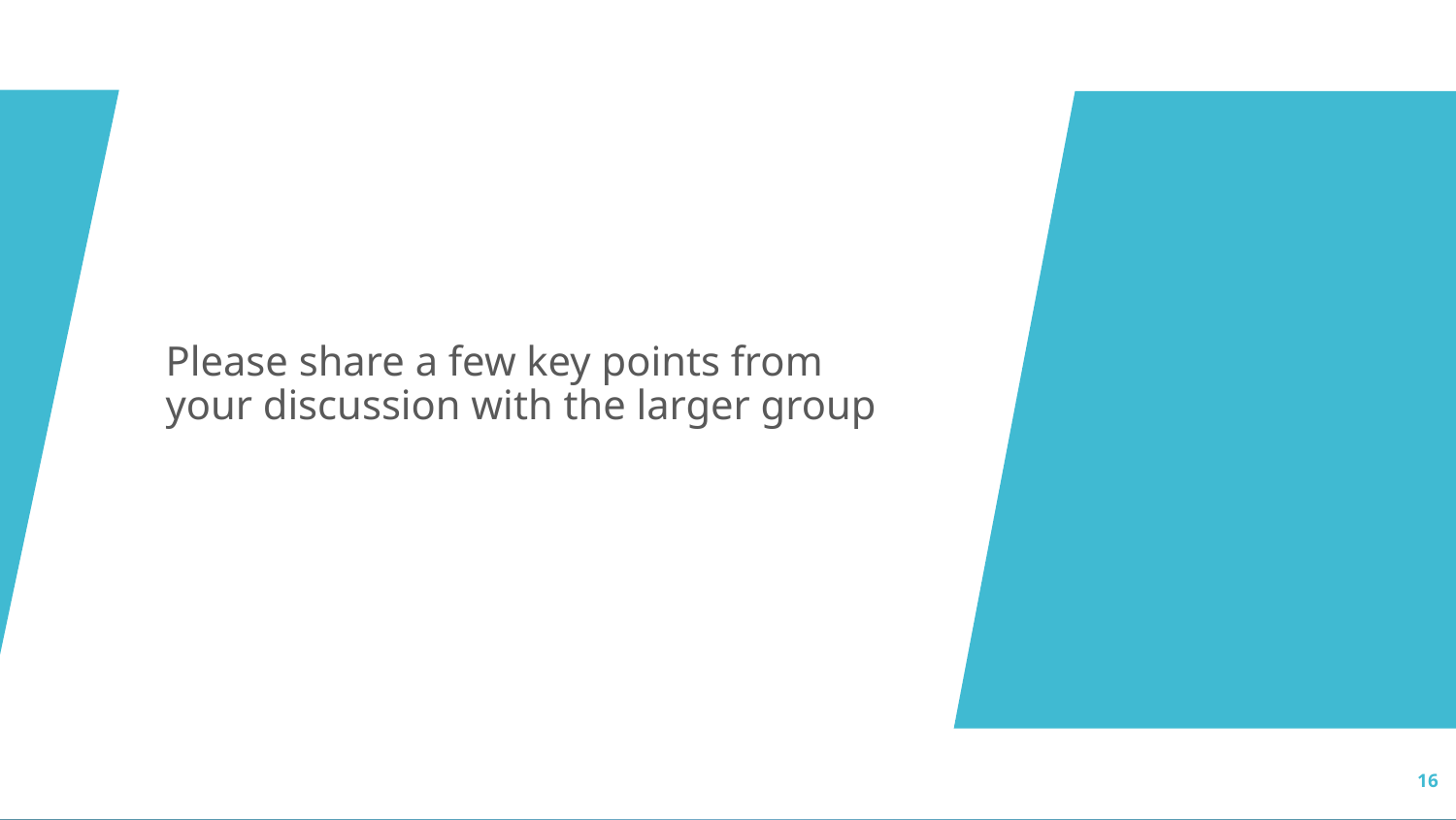

Please share a few key points from your discussion with the larger group
16

## Slide 17
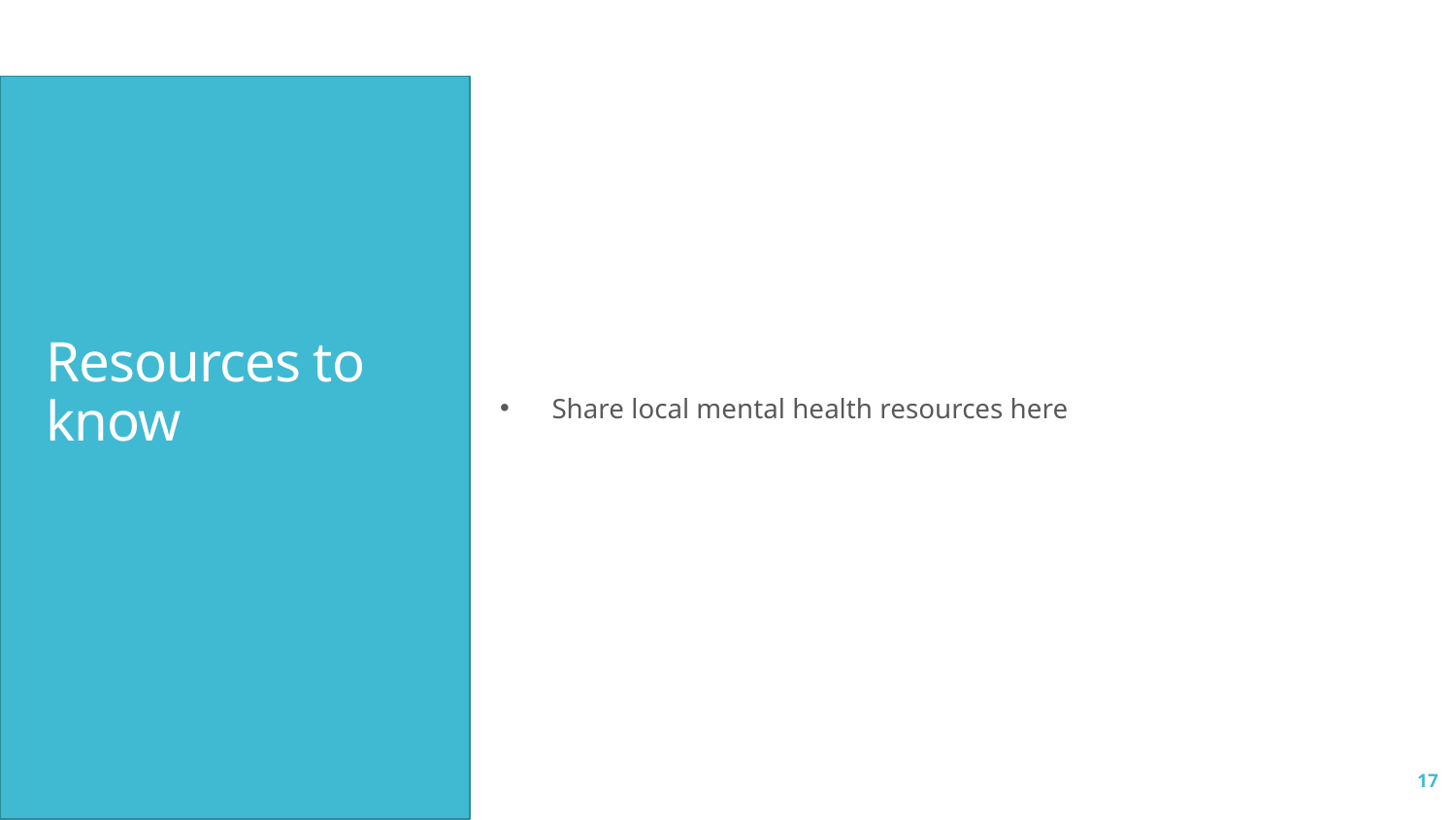

Resources to know
Share local mental health resources here
17

## Slide 18
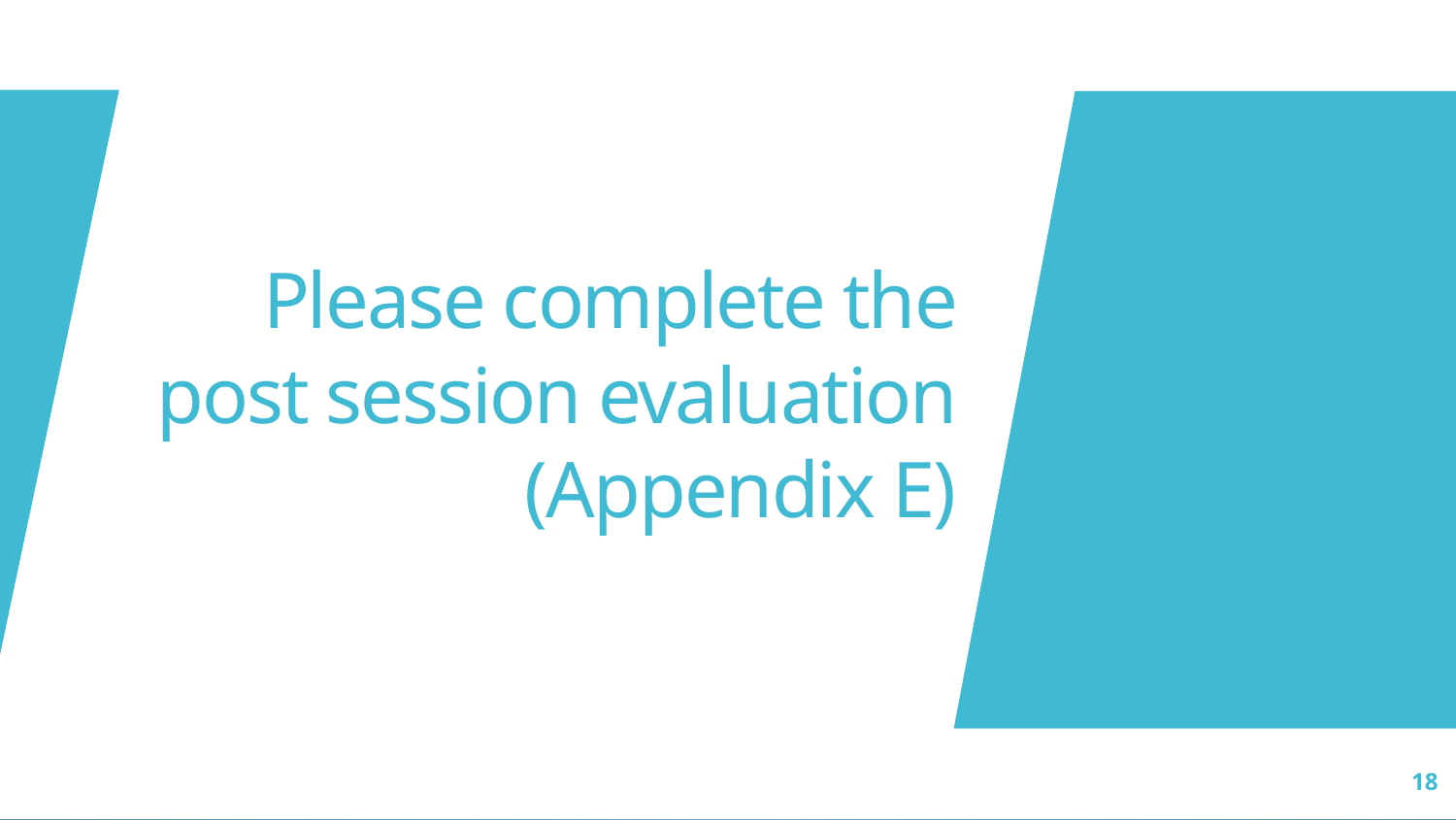

# Please complete the post session evaluation (Appendix E)
18

## Slide 19
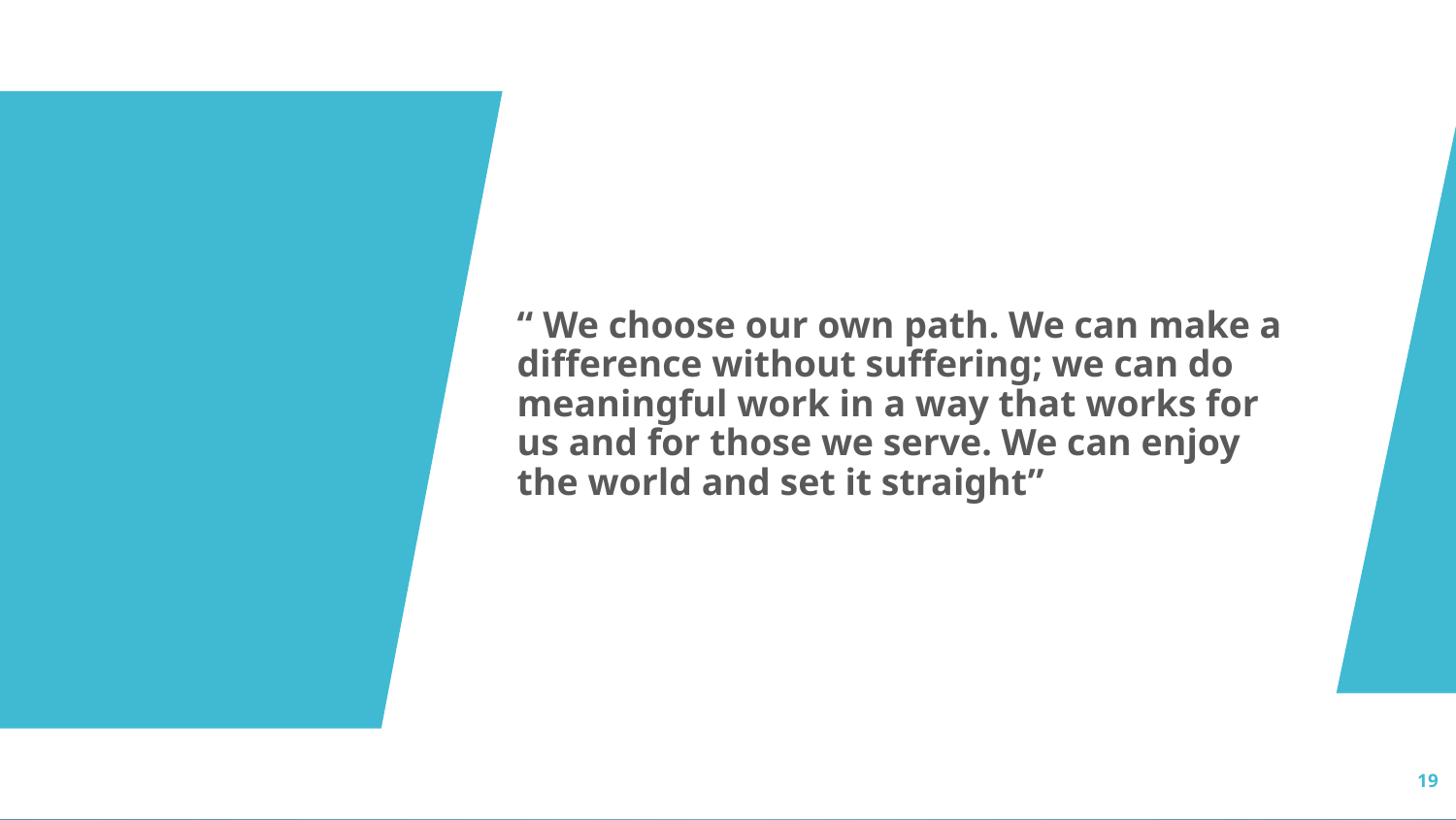

“ We choose our own path. We can make a difference without suffering; we can do meaningful work in a way that works for us and for those we serve. We can enjoy the world and set it straight”
19

## Slide 20
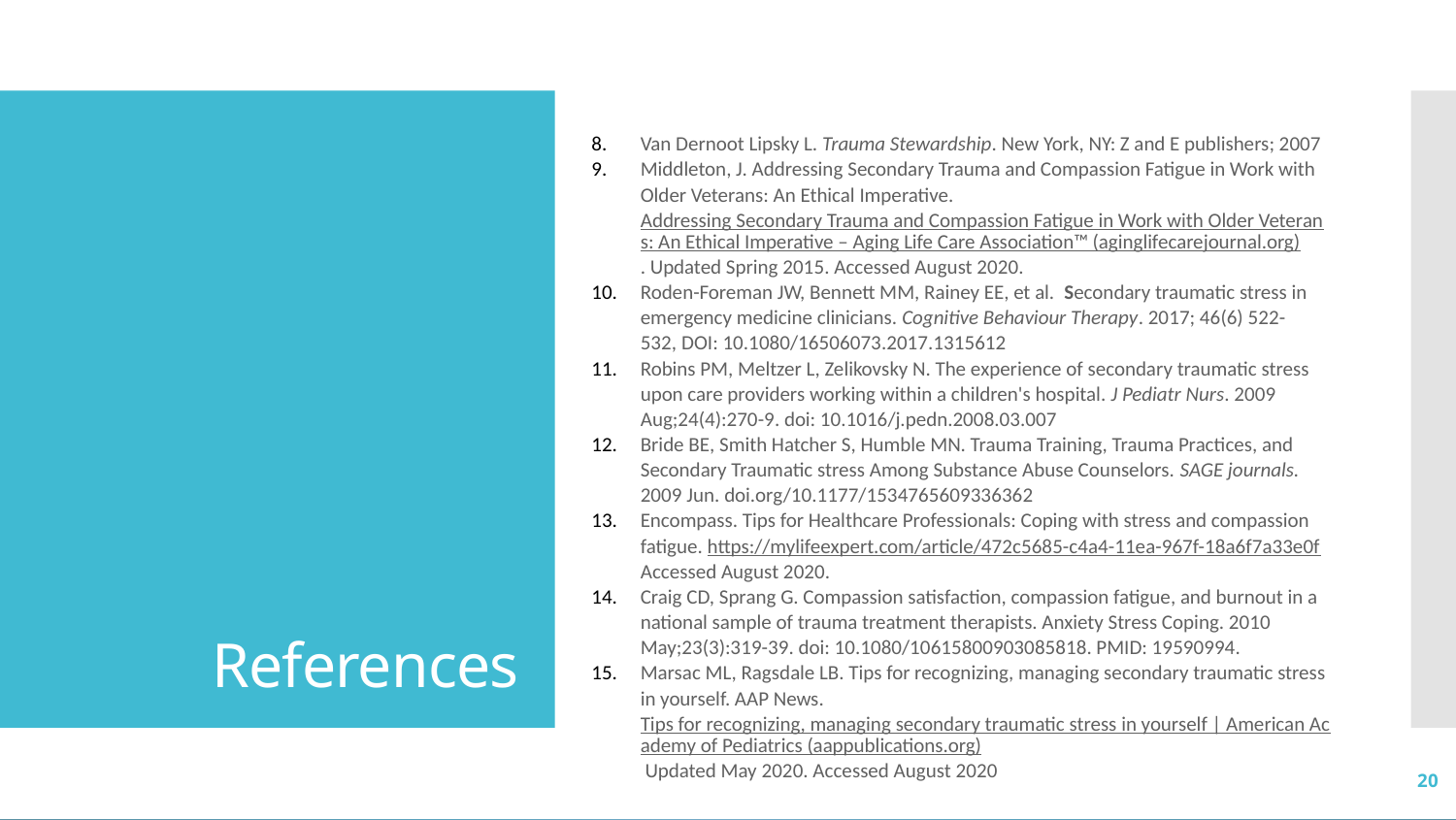

Van Dernoot Lipsky L. Trauma Stewardship. New York, NY: Z and E publishers; 2007
Middleton, J. Addressing Secondary Trauma and Compassion Fatigue in Work with Older Veterans: An Ethical Imperative. Addressing Secondary Trauma and Compassion Fatigue in Work with Older Veterans: An Ethical Imperative – Aging Life Care Association™ (aginglifecarejournal.org). Updated Spring 2015. Accessed August 2020.
Roden-Foreman JW, Bennett MM, Rainey EE, et al.  Secondary traumatic stress in emergency medicine clinicians. Cognitive Behaviour Therapy. 2017; 46(6) 522-532, DOI: 10.1080/16506073.2017.1315612
Robins PM, Meltzer L, Zelikovsky N. The experience of secondary traumatic stress upon care providers working within a children's hospital. J Pediatr Nurs. 2009 Aug;24(4):270-9. doi: 10.1016/j.pedn.2008.03.007
Bride BE, Smith Hatcher S, Humble MN. Trauma Training, Trauma Practices, and Secondary Traumatic stress Among Substance Abuse Counselors. SAGE journals. 2009 Jun. doi.org/10.1177/1534765609336362
Encompass. Tips for Healthcare Professionals: Coping with stress and compassion fatigue. https://mylifeexpert.com/article/472c5685-c4a4-11ea-967f-18a6f7a33e0f Accessed August 2020.
Craig CD, Sprang G. Compassion satisfaction, compassion fatigue, and burnout in a national sample of trauma treatment therapists. Anxiety Stress Coping. 2010 May;23(3):319-39. doi: 10.1080/10615800903085818. PMID: 19590994.
Marsac ML, Ragsdale LB. Tips for recognizing, managing secondary traumatic stress in yourself. AAP News. Tips for recognizing, managing secondary traumatic stress in yourself | American Academy of Pediatrics (aappublications.org) Updated May 2020. Accessed August 2020
# References
20

## Slide 21
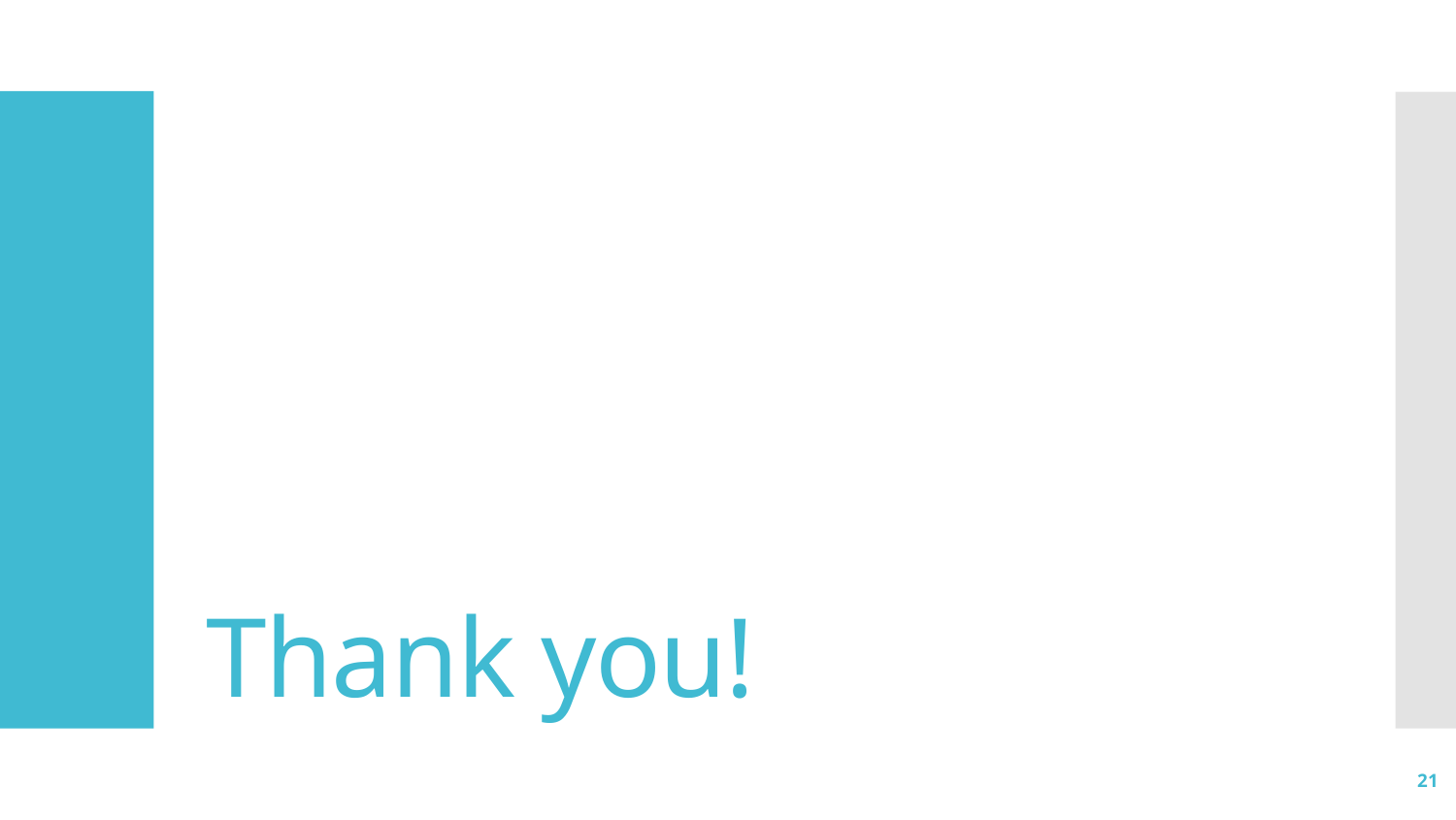

# Thank you!
21
