# Supplementary material for: Trauma Exposure Response: How Secondary Trauma Affects Personal and Professional Life
Source: MedEdPORTAL. 2021 Nov 22;17:11192. doi: 10.15766/mep_2374-8265.11192 (PMC8607743; doi:10.15766/mep_2374-8265.11192)
Supplement: Supplementary file 1 — Facilitator Guide.docxTrauma Exposure Response Presentation.pptxTrauma Exposure Response Handout.docxSmall-Group Exercises and Reflection Questions.docxPostsession Evaluation.docx [file mep_2374-8265.11192-s001.zip › C. Trauma Exposure Response Handout.docx]

**Trauma Exposure Response**

**Now that you have had a chance to learn about some principles of secondary trauma exposure, please take a moment to reflect on your experiences. Which of the following symptoms of trauma exposure response have you experienced in your medical training?** *Please take 5 minutes to review the list below*

- Feeling helpless and hopeless
- A sense that one can never do enough
- Always in a hurry/on-edge
- Difficulty sleeping
- Difficulty making decisions
- Intrusive thoughts about patients and/or ruminating about challenging cases
- Feelings of disconnection from colleagues and work teams
- Minimizing- Lose ability to empathize because we are comparing others’ suffering or putting it into a hierarchy
- Chronic exhaustion/physical ailments
- Sense of persecution – feel like we lack the ability to transform our circumstances
- Guilt
- Fear
- Anger and cynicism
- Inability to empathize/numbing
- Increase use of substances to cope
- Grandiosity – an inflated sense of importance related to one’s work. Work defines your identity

**Has some of what you have seen at work changed who you are when you are not at work? For example, are you a different parent or partner or friend due to what you have witnessed on the job?**
