# Supplementary material for: Trauma Exposure Response: How Secondary Trauma Affects Personal and Professional Life
Source: MedEdPORTAL. 2021 Nov 22;17:11192. doi: 10.15766/mep_2374-8265.11192 (PMC8607743; doi:10.15766/mep_2374-8265.11192)
Supplement: Supplementary file 1 — Facilitator Guide.docxTrauma Exposure Response Presentation.pptxTrauma Exposure Response Handout.docxSmall-Group Exercises and Reflection Questions.docxPostsession Evaluation.docx [file mep_2374-8265.11192-s001.zip › E. Postsession Evaluation.docx]

|  | **Strongly Disagree** | **Disagree** | **Neutral** | **Agree** | **Strongly Agree** |
| --- | --- | --- | --- | --- | --- |
| \| I have improved my understanding of trauma exposure response \| \| --- \| | 1 | 2 | 3 | 4 | 5 |
| I understand the signs of trauma exposure response | 1 | 2 | 3 | 4 | 5 |
| \| I recognize steps that can be taken to cope with trauma exposure response \| \| --- \| | 1 | 2 | 3 | 4 | 5 |

Trauma Exposure Response

SESSION EVALUATION

1. Please rate the value of this session
   1. Not valuable
   2. Somewhat valuable
   3. Valuable
   4. Extremely valuable
2. What was most helpful about this session?
3. How could this session be improved?
