# Supplementary material for: Trauma Exposure Response: How Secondary Trauma Affects Personal and Professional Life
Source: MedEdPORTAL. 2021 Nov 22;17:11192. doi: 10.15766/mep_2374-8265.11192 (PMC8607743; doi:10.15766/mep_2374-8265.11192)
Supplement: Supplementary file 1 — Facilitator Guide.docxTrauma Exposure Response Presentation.pptxTrauma Exposure Response Handout.docxSmall-Group Exercises and Reflection Questions.docxPostsession Evaluation.docx [file mep_2374-8265.11192-s001.zip › D. Small-Group Exercises and Reflection Questions.docx]

**Small Group Exercises/Reflection: What can we do about Trauma Exposure Response?**

Please discuss in small groups if able, or if virtual in a large group, answer questions independently below. *This activity should take approximately 25 minutes.*

**1.) Reflect on why we do what we do?**

Why do you do what you do? What keeps you going in this work? After you hear your answer remind yourself that you have the choice to do this work

2.) **Stay fully present in your experience, no matter how difficult.**

How do you stay present in your experience? Do you take time to slow down and recognize what you are going through? How could this be helpful?

**3.) Practice compassion for myself and others**

Recall a time when you were particularly hard on yourself. Ask yourself what your deepest fear was at that time. Close and replay the situation in your mind, imagining how you could have responded to yourself more compassionately. Notice how this shift in response feels.

4**.) Practice Gratefulness**

Some of our work can give us a powerful perspective on what we have to be grateful for, or can be an impetus for “post-traumatic growth”—a new appreciation for life, newfound meaning, shifts in how we view ourselves or how we relate to others, or emotional/spiritual transformation of some kind that can emerge over time after experiencing a trauma. Have your exposures to trauma at work led to growth in any surprising ways?
